# Advances in Proteomic Techniques for Cytokine Analysis: Focus on Melanoma Research

**DOI:** 10.3390/ijms18122697

**Published:** 2017-12-13

**Authors:** Helena Kupcova Skalnikova, Jana Cizkova, Jakub Cervenka, Petr Vodicka

**Affiliations:** 1 Laboratory of Applied Proteome Analyses, Institute of Animal Physiology and Genetics, Czech Academy of Sciences, Rumburska 89, 27721 Libechov, Czech Republic; cizkova@iapg.cas.cz (J.C.); cervenka@iapg.cas.cz (J.C.); vodicka@iapg.cas.cz (P.V.); 2 Department of Veterinary Sciences, Faculty of Agrobiology, Food and Natural Resources, Czech University of Life Sciences, Kamycka 129, 16500 Prague, Czech Republic; 3 Department of Cell Biology, Faculty of Science, Charles University, Vinicna 7, 12843 Prague 4, Czech Republic

**Keywords:** cytokine, cancer, melanoma, secretome, proteomics, T-cell, biomarker, ultrasensitive, immunoassay, mass spectrometry

## Abstract

Melanoma is a skin cancer with permanently increasing incidence and resistance to therapies in advanced stages. Reports of spontaneous regression and tumour infiltration with T-lymphocytes makes melanoma candidate for immunotherapies. Cytokines are key factors regulating immune response and intercellular communication in tumour microenvironment. Cytokines may be used in therapy of melanoma to modulate immune response. Cytokines also possess diagnostic and prognostic potential and cytokine production may reflect effects of immunotherapies. The purpose of this review is to give an overview of recent advances in proteomic techniques for the detection and quantification of cytokines in melanoma research. Approaches covered span from mass spectrometry to immunoassays for single molecule detection (ELISA, western blot), multiplex assays (chemiluminescent, bead-based (Luminex) and planar antibody arrays), ultrasensitive techniques (Singulex, Simoa, immuno-PCR, proximity ligation/extension assay, immunomagnetic reduction assay), to analyses of single cells producing cytokines (ELISpot, flow cytometry, mass cytometry and emerging techniques for single cell secretomics). Although this review is focused mainly on cancer and particularly melanoma, the discussed techniques are in general applicable to broad research field of biology and medicine, including stem cells, development, aging, immunology and intercellular communication.

## 1. Introduction to Melanoma and Cytokines

### 1.1. Melanoma

Melanoma is one of the ten most common types of cancer with a steadily increasing incidence. In Europe, 100,300 of the new cases and 22,200 of deaths from melanoma arose in 2012 [1]. In United States melanoma belongs to the 5 most prevalent cancers in males [2].

Melanoma results from malignant transformation of melanocytes, which are naturally occurring pigmented cells in the epidermis. Melanocytes are responsible for the production of an endogenous pigment melanin, protecting the skin from harmful ultraviolet radiation [3]. Melanocytes originate from neural crest. Outside the skin, melanocytes occur in eye, mucosal epithelia and meninges [4] and therefore melanomas can be divided according to the place of origin to the most common cutaneous melanoma, uveal melanoma and mucosal melanoma [5]. Factors influencing the development of melanoma include: the degree of skin pigmentation, the length of skin exposure to Ultraviolet (UV) radiation [6,7] and hereditary predisposition, as about 10% of melanomas have familial occurrence [8].

To study melanoma pathogenesis and facilitate development of targeted therapies, several animal models have been developed, including mouse, pig, horse, dog, zebrafish, chick embryo (to study neural crest cell migration) and others (reviewed in [9,10,11,12]).

### 1.2. Spontaneous Regression

Melanoma belongs to the tumours with the highest rate of documented spontaneous regression [13]. Spontaneous regression is the partial or complete disappearance of a malignant tumour in the absence of anticancer treatment. Melanoma regression is mostly partial, with rare reports of complete regression (38 documented cases in 2005 [14]). Melanoma regression occurs probably in 3–5% of tumours [15,16], although regression rates up to 50% have been documented [17]. Such a large variation in reported regression prevalence may be caused by various evaluation criteria used among studies [18]. Nonetheless, regression occurs more frequently in early stages of tumours (Breslow’s thickness bellow 1.5 mm) [15] and is very rare (0.23%) in melanoma metastases [17].

Spontaneous regression is frequently accompanied by tumour infiltration by CD8+ and also CD4+ T-cells [18] and vitiligo, a hypopigmented skin lesion, which may be associated with an antitumour response to melanocytes [19]. Interestingly, in patients with multiple asynchronous melanomas, the incidence of spontaneous regression was higher in successive melanomas compared to primary tumour, suggesting an increased anti-cancer immunity evoked by the primary lesion [20].

As the immune system plays an active role in melanoma regression, a strong evidence is provided that melanoma would be amenable by immunotherapies [19].

### 1.3. Melanoma Treatment and Immunotherapies

While early stages of melanoma can be treated by surgical excision, tumours in later stages with vertical growth and metastasis are refractory to therapies. The tumour excision can be supplemented with chemotherapy and/or immunotherapy, as melanoma is typically an immunogenic tumour [16]. First cytokine therapies of melanoma were realized in the 1980s with interleukin 2 (IL-2) and interferon α (IFNα) and up to 20% of tumours showed response to such therapies. However, the therapy was often accompanied by toxic effects, including flu-syndrome, gastrointestinal symptoms and weigh gain but also more serious symptoms like multi organ (heart, lung, liver, kidney and central nervous system) toxicity [21,22,23,24].

Nowadays, better therapeutic responses with a lower incidence of side effects are achieved by combination of cytokine application with other approaches, e.g., monoclonal antibodies [25,26], kinase inhibitors [27], tumour-infiltrating T-cell [28], chemotherapeutic agents [29], stereotactic body radiotherapy [30] or peptide vaccines [31,32]. The overall survival of advanced-stage melanoma patients has improved dramatically in the last 5 years with development of immunotherapy by monoclonal antibodies blocking cytotoxic T-lymphocyte-associated antigen 4 (CTLA-4) and programmed cell-death protein 1 (PD-1) and with application of B-Raf or MEK kinase inhibitors in BRAF gene mutant tumours [33]. Despite the progress in melanoma treatment, the efficacy of immunotherapies is still unpredictable and many patients eventually develop resistance to treatment [33].

### 1.4. Tumour Microenvironment

Cells in tissues are living in precisely defined microenvironments, called also the niche or the stroma. Similar to adult tissue stem cells, where the microenvironment is essential for stemness maintenance, the environment in malignant tumours is indispensable for control of the cancer stem cell division and tumour growth [34,35]. Melanoma microenvironment is formed not only by intrinsic malignant cells but also by complex interactions with other cells, such as keratinocytes, cancer-associated fibroblasts (CAFs), immune cells, blood/lymphatic endothelial cells as well as their extracellular products, that all participate in tumour formation and growth [34,36]. To characterize the tumour microenvironment, Paulitschke et al. analysed proteins secreted by individual cell types using shotgun proteomics [37]. Secretomes of normal skin human fibroblasts, CAFs from mouse melanoma, primary human melanocytes, M24met melanoma cells, human umbilical vein endothelial cells (HUVECs), monocyte-derived dendritic cells and cancer-associated fibroblasts isolated from bone marrow of multiple myeloma patients have been compared. Several extracellular matrix proteins and matrix remodelling enzymes have been commonly identified across samples. Macrophage migration inhibitory factor (MIF) was identified in all samples except of the human fibroblast secretome. Moreover, secretion of Interleukin-20 (IL-20), a structural protein of melanosomes Melanocyte protein Pmel 17, Melanoma antigen gp75 and Melan-A protein were typical for primary melanocytes [37].

Crosstalk between keratinocytes and melanocytes exists already in a healthy skin. After UV irradiation, keratinocytes produce the α-melanocyte stimulating hormone (αMSH) that stimulates melanocytes to produce melanin. Melanin-containing vesicles (melanosomes) are then transferred to keratinocytes, where the pigment protects keratinocyte nuclei from UV-caused DNA damage [4,38]. In melanoma, epidermis surrounding the tumour exhibits increased thickness. Transcriptomic study identified Basic fibroblast growth factor (bFGF), GRO1 oncogene (GROα, CXCL1), IL-8 and Vascular endothelial growth factor A (VEGF-A) to participate in such an effect of melanoma cells on keratinocytes [39].

Fibroblasts are major cells in the connective tissue and are main producers of extracellular matrix components. In healthy skin, fibroblasts also produce factors regulating proliferation, differentiation and survival of melanocytes (e.g., Stem cell factor (SCF), bFGF, Hepatocyte growth factor (HGF), Transforming growth factor-β (TGFβ)) or stimulating melanogenesis (Keratinocyte growth factor (KGF), Neuregulin-1 (NRG-1)) (reviewed in [40]). Normal fibroblasts are also capable of secretion of IL-6, IL-8 and GROα and the interleukin 6 and 8 release significantly increases in presence of either keratinocytes or cancer cells (FaDu hypopharyngeal carcinoma epithelial cells) [41]. Cancer-associated fibroblasts are frequent cells in tumour stroma. *In vitro* co-culture experiments show that CAFs promote migration and invasiveness of melanoma cells and such migration is dependent on IL-6 and IL-8 secretion. Application of antibodies blocking the IL-6 and IL-8 activity fully inhibits the melanoma cell migration *in vitro* [42]. Increased IL-6 and IL-8 expression have previously been well documented to correlate with tumour progression (reviewed in [43,44]). CAFs from melanoma influence also keratinocytes and, among others, induce expression of keratin type 14 (marker of proliferating basal layer keratinocytes) and vimentin (marker of epithelial-to-mesenchymal transition) in keratinocytes [45].

Among the immune cells infiltrating tumour, the T-lymphocytes play a central role in anti-cancer immunity and are thus in main focus of melanoma immunotherapies. The degree of T-cell infiltration and T-cell phenotype in the tumour are important predictors of response of patients to cancer immunotherapy [46]. An effort is applied to the search for melanoma antigen-specific cytotoxic T-cells that could be used in therapy [47]. Adoptive cell therapy with tumour infiltrating T-lymphocytes, isolated from patient’s tumour, *in vitro* expanded and applied via infusion, is already showing positive outcomes as an effective treatment for metastatic melanoma [48]. On the other hand, tumour infiltration by immunosuppressive cells, such as regulatory T-cells (Tregs) or immunosuppressive tumour-associated macrophages (M2 TAMs), secreting anti-inflammatory cytokines, such as TGFβ and IL-10 and pro-angiogenic factors, or expressing a PD-ligand, relates to unfavourable prognosis. Such immunosuppressive cells represent targets of potential immunotherapies [49,50,51]. Other immune cells present in tumour stroma, such as natural killer (NK) cells [52], plasmacytoid dendritic cells [53], B-lymphocytes [54] or others, are less investigated. Nonetheless, immune cell components of malignant melanoma could highlight new predictive biomarkers for response to immunotherapy and indicate new immunotherapeutic approaches [51].

Extracellular products are fundamental parts forming the tumour microenvironment. Not only cellular interactions with extracellular matrix but also enzymes (e.g., matrix remodelling proteases), secreted factors (including cytokines, chemokines, growth factors, angiogenic factors, etc.), extracellular vesicles (EVs), such as exosomes [55,56], EV transferred miRNAs [57], nutrient and oxygen availability [58] and other factors participate in control of tumour progression.

Therapeutic manipulation of tumour microenvironment seems to be a highly promising approach in cancer therapy [35].

### 1.5. Cytokines

Cytokines are proteins that participate in cell signalling, intercellular communication and in many cellular and immunological functions. Cytokines are produced by a broad range of cells but in oncological research the most attention is paid on cytokines produced by immune cells. Cytokines exert various functions from regulation of inflammatory response, through regulation of cell growth, differentiation, chemotaxis, angiogenesis and many others. From analytical point of view, cytokines represent mostly small proteins (peptides), however, the molecular mass can cover ranges from approximately 6 to 70 kDa [59].

In cancer, cytokines represent key regulators that promote migration, invasion and metastasis of cells. The expression and activity of cytokines are deregulated in many cancer types [60]. Transformed cells produce pro-inflammatory cytokines, chemokines and growth factors that support cell survival and proliferation and promote inflammation and angiogenesis. This results in recruitment of immune and stromal cells into the tumour. Mediators secreted by the growing tumour, including cytokines, further contribute to the cell proliferation, angiogenesis and inflammation but also to a matrix remodelling, adhesive molecule expression changes and increased vascular permeability, leading to a formation of metastatic microenvironment [60,61,62].

Diagnostic potential and prognostic significance of cytokines in cancer have already been documented. Interleukin 8 is recognized as a chemotactic factor for neutrophils, however, it possesses additional functions in angiogenesis and matrix-metalloproteinase activation. Angiogenesis and metastases of melanoma may be accompanied by secretion of IL-8 from tumour stroma together with its signalling through CXCR2 receptor [62,63]. Serum levels of IL-8 correlate with tumour stage [64] and IL-8 has been suggested as a circulating biomarker of melanoma [65]. Similar to IL-8, production of HGF by stromal cells and activation of Met receptor by HGF, influences melanoma invasiveness. Elevated HGF levels in blood as well as presence of Met-containing exosomes are connected to melanoma metastases and resistance to therapy [66,67]. Chemokines CCL17 (Thymus and activation regulated chemokine (TARC)) and CCL22 (C-C motif chemokine 22) produced by tumour infiltrating macrophages may help to recruit Tregs to tumour and maintain an immunosuppressive tumour microenvironment in melanoma [50]. Another study analysed cytokine profile of cerebrospinal fluid in brain metastasis of melanoma. Elevated levels of IL-8, Macrophage inflammatory protein-1β (MIP-1β), Interferon gamma-induced protein 10 (IP-10) and TARC, have been reported and such molecules represent potential metastasis predicting biomarkers [68].

In a study of therapeutic effects of ipilimumab (monoclonal antibody targeting CTLA-4), an increased secretion of IL-1β, IL-2, IL-4, IL-5, IL-7, IL-8, IL-10, IL-13, IL-17, Granulocyte-colony stimulating factor (G-CSF), Granulocyte-macrophage colony-stimulating factor (GM-CSF), HGF, IFNγ, Monocyte chemoattractant protein 1 (MCP-1, CCL2) and VEGF from peripheral blood mononuclear cells (PBMCs) showed a trend towards better recurrence free survival in melanoma [69]. Interestingly, these cytokines belong to various functional groups with pro-inflammatory and anti-inflammatory effects [69]. Possibilities of therapeutic administration of cytokines to induce anti-tumour responses in melanoma have been reviewed by Xu et al. [70]. Cytokine employment to melanoma treatment is currently mostly limited to IL-2 [71] and IFNα [72], although attempts of application of GM-CSF encoded by oncolytic virus have been done [73]. Detecting the number of individual cytokines is useful for planning of targeted therapeutic approaches for melanoma or it can serve as an important element in evaluating of the effectiveness of an ongoing treatment.

The purpose of this review is to give an overview of recent advances in techniques for the detection and quantification of cytokines at the protein level, applicable to melanoma research. Techniques covered span from single molecule to multiplex assays, including emerging ultrasensitive techniques and analysis of individual cytokine producing cells. Developments in technologies of detection from colour enzymatic reaction through chemiluminescence, fluorescence to ultrasensitive antibody detection coupled to PCR amplification, surface plasmon resonance and other electrochemical, optical or mechanical immunosensing led to sensitive detection of picogram to even femtogram per millilitre values. In this review, the term cytokine is used in a broader meaning and covers also chemokines, growth factors, angiogenic factors and other factors, as levels of such molecules reach similarly low concentrations and such molecules often possess a pleiotropic effect that may participate in regulation of similar cellular processes. Although this review is focused mainly on cancer and particularly melanoma research, the discussed techniques are in general applicable to broad research field of biology/medicine, including stem cells, development, degeneration, immunology and inter-cellular communication and signalling.

## 2. Cytokine Detection Techniques

Cytokines are key players in inter-cellular communication and immune system regulation and an extensive effort is applied on cytokine research in cancer. Significant development of proteomic techniques achieved in the last 10 years led to establishment of powerful tools for cytokine detection and quantification. However, accurate and reproducible measurement of cytokines faces several challenges. These include low cytokine concentrations (reaching picograms per millilitre), dynamics in cytokine secretion in time (transient secretion), cytokine binding to other proteins in the sample or interference with other proteins/antibodies in the assay [74]. Moreover, the biological effect may result from a large number of cytokines that act in complicated regulatory networks [74,75], which places demands not only on simultaneous detection of multiple cytokines but also may lead to convoluted interpretation of proteomic results. Current proteomic techniques for cytokine analysis are based on mass spectrometry and immunoassays.

### 2.1. Mass Spectrometry

Mass spectrometry (MS) is a fundamental technique in proteomics, applicable for protein identification and quantification. In a classical bottom-up proteomic workflow, proteins are isolated (enrichment or prefractionation step can be added) and digested by specific protease (e.g., trypsin) to smaller peptides. Peptides are then concentrated and desalted, separated by high-performance liquid chromatography (HPLC), ionised and analysed by MS. Tandem mass spectrometer (MS/MS) measures exact mass-to-charge (*m*/*z*) ratio of peptides (precursor ions) in MS experiment and their fragment ions in MS/MS experiment. This approach allows very specific identification and quantification of peptides/proteins.

Despite being a powerful proteomic technique, application of MS to cytokine analysis is challenging. Several limitations of MS should be considered: (1) MS is able to detect only charged (ionised) peptides but not all peptides are ionised with the same efficiency. Moreover, proteins with low molecular weight (including most cytokine molecules) provide less peptides and taken together with low abundance, these peptides will not be detected without enrichment or prefractionation [76]. (2) Sensitivity of shotgun MS is usually insufficient for detection of low abundant proteins (such as cytokines) in complex biological samples (blood serum or plasma) without employment of sample depletion or prefractionation. For example, the concentrations of serum albumin and IL-6 in human plasma differ by 10 orders of magnitude [77]. (3) MS-based approach is highly reproducible but quality controls (e.g., internal standard proteins/peptides) are essential for control of protein digestion efficacy, HPLC peptide separation and MS performance. Shotgun proteomics (data dependent acquisition (DDA), see below) is partially stochastic, because precursor ions are selected for fragmentation by computer-controlled algorithm according to ion signal intensities, thus slightly different lists of identified proteins may yield from repeated analysis of the same complex sample, especially in case of low abundant proteins. On the other hand, targeted methods (selected reaction monitoring (SRM) or multiple reaction monitoring (MRM), see below) do not suffer this issue and offer robust and reproducible analysis [76]. (4) Protein identification depends on protein databases and may suffer from insufficient coverage for non-human organisms (e.g., animal models used in biomedical research). As an example from our experience, the Swiss-Prot database (UniProt Release 2017_10) contains 1421 reviewed proteins for pig (*Sus scrofa*), compared to 20,239 reviewed proteins for human (*Homo sapiens*). Although there is a possibility to use reference proteome based-on genome assembly to get deeper coverage, data has to be evaluated carefully, as protein duplications or errors in protein sequence occur.

Taken together, MS approaches provide relatively easy multiplexing capability and higher specificity but lower sensitivity than immunoassays.

#### 2.1.1. Data Dependent Acquisition (DDA)—Shotgun Proteomics

Shotgun proteomics aims to identify all (detectable) proteins that are present in a sample without prior knowledge or pre-selection of analytes. Shotgun proteomics requires use of tandem mass spectrometers and the method works in cycles. Firstly, a scan for precursor ions is done (MS experiment). Then a predefined number of precursor ions with the highest signal intensity is automatically selected for fragmentation. The resulting fragment ions are then analysed in a MS/MS experiments. When all precursor ions meeting selection criteria are fragmented and analysed, new cycle starts. Depending on the sample, tens to thousands of proteins can be identified or quantified by such an approach. However, due to partial stochasticity of ion selection for fragmentation, it is necessary to verify quantification results of DDA measurement by an independent method (e.g., western blot).

Although shotgun proteomics is capable of detecting analytes in the femtomole or attomole range, its sensitivity is mostly insufficient for detection of cytokines in blood plasma [77]. However, cytokines might still be detectable by MS in culture media conditioned by cells *in vitro*, as the culture medium (particularly a serum-deprived formula) represents less complex sample and as the cytokine production *in vitro* may be higher (due to more or less uniform cell populations cultured in a limited volume) and more synchronized (e.g., by cell activation). Nonetheless, the cytokine production by cultured cells may not fully resemble the *in vivo* physiological state.

Shotgun analysis of secretomes of melanoma cell lines from three different metastases of the same patient was performed by Rocco et al. [78]. Cytokine Growth/differentiation factor 15 (GDF15) was identified in all three secretomes. Moreover, Melanoma-derived growth regulatory protein (MIA) and Osteopontin (SPP1) were identified in secretome of one particular metastatic cell line [78]. Several cytokines, including GDF15, IL-6, IL-8, IL-18 and MIA, have been identified by shotgun MS in a study of secretomes of lung metastases derived from melanoma and breast cancer [79]. Interestingly, the GDF15, also called Macrophage Inhibitory Cytokine-1 (MIC-1), has been previously recognized as a promising biomarker of metastatic melanoma [80].

#### 2.1.2. Data Independent Acquisition

Data independent acquisition (DIA), in contrast to DDA, provides information about quantity of all detected proteins, without specific selection of certain proteins (peptides) for analysis. DIA, for example Sequential Windowed Acquisition of all Theoretical Mass Spectra (SWATH-MS) method, is used for a discovery-based approach. All precursor ions in a specific window of *m*/*z* values in MS experiment are fragmented together and these fragments are detected in MS/MS experiment. This approach allows for identification and quantification of peptides, which would not be selected for fragmentation and thus remained undetected in DDA analysis. Assay library is essential for identification of peptides from such complex spectra from MS/MS experiments. The library is generated by shotgun measurements of the same samples, or for deeper coverage of proteome, assay library can be prepared by measurements of many samples of different cell types, tissues or sample fractionations. Human proteome assay library containing more than 10,000 proteins is publicly available [81]. SWATH-MS approach was shown to be reproducible with high selectivity, accuracy and sensitivity [82]. Capability of SWATH-MS to serve as a tool in search for biomarkers, even in a clinical research, was reviewed recently [83].

The DIA method enabled identification of several growth factors and cytokines in medium conditioned by malignant cells. Lin et al. analysed glycoproteins in secretomes of colon adenocarcinoma cell line and its derived metastatic cells [84]. They identified 568 proteins in total, with 421 proteins suitable for quantification, including cytokines such as GDF15, Insulin-like growth factor II (IGF-II), Macrophage colony-stimulating factor 1 (M-CSF) and TGFβ-1. Study of secretomes from malignant mesothelioma cell lines and mesothelial cells revealed 421 secreted proteins including M-CSF, MIF and TGFβ-1 [85].

#### 2.1.3. Selected Reaction Monitoring and Multiple Reaction Monitoring

**Figure 1:**
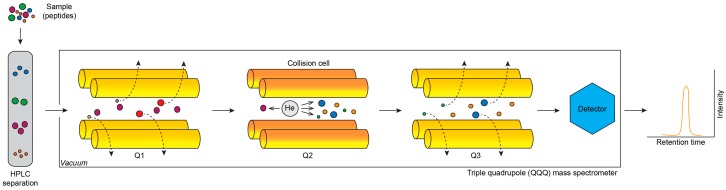
Principle of SRM assay for targeted protein quantification by mass spectrometry. Peptides from sample are separated by reversed-phase HPLC and eluted with specific retention times. Ionised peptides are analysed by triple quadrupole (QQQ) MS instrument. In quadrupole Q1, particular peptide with preset *m*/*z* value is selected (precursor ion, purple colour) and passed to Q2, whereas other ions are filtered out (dashed line arrows). In Q2, fragmentation of the selected precursor ion occurs, due to collisions with molecules of inert gas (helium—grey colour). Originating fragment ions are filtered by Q3 (dashed line arrows) and only fragment ion with preset *m*/*z* value (yellow colour) is passed to detector, where fragment ion intensity is recorded. As a preset pair of precursor and fragment ion with particular *m*/*z* (transition) is measured, the SRM method is very specific.

SRM and MRM approaches have been developed for targeted analysis and quantification of selected protein(s) in a complex sample. Such approaches rely on a specific tandem MS instrument—triple quadrupole (QQQ). First quadrupole (Q1) selects precursor ions of particular *m*/*z*, which are then passed to the second quadrupole (Q2) that serves as a collision cell, where precursor ions are fragmented by collisions with molecules of inert gas (e.g., helium). The third quadrupole (Q3) then selects specific type of fragment ions of particular *m*/*z* (Figure 1). The first and third quadrupole thus works as *m*/*z* filters analysing predefined pair of precursor and fragment ions, so-called transition. This approach cannot be used for discovery analyses, because this method enables measurement of predefined transitions only. Specificity of the measurement is assured not only by Q1 and Q3 but also by elution of peptides from HPLC system (retention time). Addona et al. proved that MRM approach with standardized protocol is robust and reproducible between different laboratories and MS platforms [86].

MRM method is capable to absolutely quantify 142 proteins (312 peptides) from non-depleted and non-enriched human plasma in concentration ranges 31 mg/mL to 44 ng/mL in one 43 min analysis [87]. Interestingly, purchase of a MS instrument for MRM measurements represents approximately same expense as a development of five new ELISA assays [88].

In focus on low abundant proteins, Bredehöft et al. developed MRM method for quantification on human IGF-1 after its immunoaffinity isolation from plasma samples with detection limit 20–50 ng/mL [89]. Anderson et al. used Stable Isotope Standards and Capture by Anti-Peptide Antibodies (SISCAPA) approach to purify peptides of IL-6 and tumour necrosis factor-α (TNFα) from human plasma samples and employed SRM for their precise quantification [90]. The SISCAPA method was later used for quantification of other cytokines (e.g., G-CSF and Interleukin-1 receptor antagonist (IL-1ra)) with detection limits approximately 1 ng/mL in human plasma [91].

#### 2.1.4. Immunoassays Coupled with MS Analyses

Sherma et al. described Mass Spectrometric Immunoassay (MSIA) approach for detection and quantification of MIF in human serum samples [92]. MIF was firstly enriched by MIF antibodies and then measured by MALDI-TOF (Matrix-assisted laser desorption ionisation-Time-of-flight) MS instrument. This method allows for quantification of MIF in concentration 1.56–50 ng/mL with a good correlation to ELISA. Moreover, different proteoforms of MIF (posttranslationaly modified, e.g., by cysteinylation) could be identified and quantified [92].

Direct immunoaffinity desorption/ionization (DIADI) MS is a recently developed method involving affinity capture of analytes by specific antibodies on protein chip and detection/quantification by MS instrument [93]. Protein chips (e.g., antibodies, lectins etc.) are prepared by ambient ion landing on MALDI plate. Samples are then added on the MALDI protein chip, covered with matrix and analysed by MALDI MS instrument. For example, detection limit for leptin in serum is 160 ng/mL. Performance of this approach was proved by detection of haptoglobin in human serum from 116 patients [94]. This method allows to distinguish two isoforms of haptoglobin with affinity to the same antibody.

### 2.2. Immunoassays for Quantification of Secreted Cytokines

**Figure 2:**
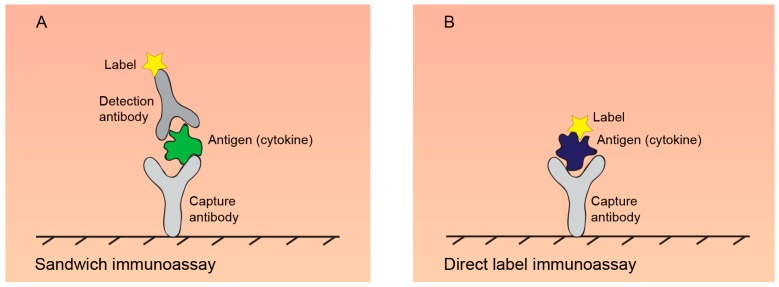
Principles of common immunoassays. (**A**) Sandwich immunoassays use matched pair of capture and detection antibody. Capture antibody is immobilized on a solid support, such as in 96-well plate, on beads, glass or a membrane array. Detection antibody conjugated to an enzyme, fluorescent tag or DNA tag, enables the quantification of cytokine; (**B**) in direct label assays, the proteins in analysed sample are labelled by fluorescent tags and incubated with an array of antibodies.

Proteomic techniques for cytokine detection/quantification rely mainly on immunoassays. Immunoassays take advantage of high sensitivity of antibodies to detect proteins of interest. In the detection of cytokines secreted to body fluids or cell culture media, common immunoassays mostly reach sensitivity in 1–100 pg/mL protein concentrations that is 10^9^ times lower than concentration of the most abundant blood plasma proteins. Sandwich immunoassays in various formats are mostly used for secreted cytokine quantification (Figure 2).

Quantification of cytokine levels by immunoassays usually depends on calibration curves, constructed from signal intensities of known standard concentrations. The availability of epitopes and specific/nonspecific binding of antibodies to antigens are strongly influenced by the sample composition (e.g., presence of other proteins, ions, salts, pH, or sample viscosity). Minimizing of such matrix effects, e.g., by resembling the sample matrix composition (sample background) also in calibration standards, or by sample dilution, is a prerequisite for low assay background and proper quantification. Moreover, suitable controls, e.g., negative, positive, in-house quality controls (sample that has been previously measured) or even spike-in controls should be included to validate assay performance. Following sections list immunoassays in the order based on the level of multiplexing, i.e., the number of simultaneously measurable analytes.

#### 2.2.1. ELISA

ELISA (enzyme-linked immunosorbent assay), developed in 1971 [95,96], is a crucial technique for single analyte quantification in basic research and also in clinical settings. The sandwich ELISA, due to combination of two specific antibodies, enable analysis of complex samples with high specificity for analyte detection without the need of sample pre-fractionation [97]. The sample is incubated with a capture antibody immobilized in 96-well plate. Then, the sample is washed out and the detection antibody is added. Detection antibody can be directly conjugated to an enzyme, or additional secondary detection antibody-enzyme conjugate is used. After addition of substrate, the enzyme produces a coloured product that is detected in the microplate reader. The capture and detection antibodies must be validated to work together (to bind to different epitopes on the antigen). In cytokine analysis, the commercial ELISA ready-to-use kits with optimized antibody pairs are available for broad spectrum of targets and species.

ELISA immunoassays are quite popular because of their high specificity, sensitivity, rapid turnaround time, convenience, the ease of performance and a relatively low cost [98]. However, in the need of multiple analyte measurement, running of multiple ELISAs places substantial demands on time, costs and sample amounts. The multiplex techniques may overcome such issues.

#### 2.2.2. Western Blot

Despite being developed almost 40 years ago [99], western blotting still represents a key and powerful biochemical technique for protein detection and relative quantification. Unlike ELISA, which is suitable for cytokine detection in solution, western blotting is preferably suited for analysis of intracellular cytokines and cytokines in complex biological samples and tissues. Western blotting is based on separation of denatured proteins in polyacrylamide gels followed by protein transfer to nitrocellulose or polyvinylidene difluoride membranes and detection by specific antibodies. Although the western blotting may not be as sensitive as ELISA in cytokine detection, it represents irreplaceable technique that provides additional information of protein molecular weight. Thus, western blotting can be applied to distinguish splice variants or detect cytokine molecule degradation and can also distinguish inactive precursors from the active forms. For example, IL-1β, a pro-inflammatory and pleiotropic cytokine abundant in tumours, is produced as 31 kDa precursor that is further processed by caspase-1 to a mature active 17 kDa form [100]. Western blotting enables to distinguish processed and unprocessed caspase-1 and/or IL-1β molecules and indirectly provides information about protein activation [101,102].

Western blotting can be also applied to study protein phosphorylation using phospho-site-specific antibodies. For instance, such approach was applied to study VEGF receptor phosphorylation in uveal melanoma cell lines to monitor effects of VEGF-A and its inhibitor (bevacizumab) on cell proliferation, migration and invasion and production of other cytokines [103]. Protein phosphorylation and intracellular signalling pathways can be effectively studied by multiplex techniques and various kits are offered on market.

Techniques for subcellular fractionation prior cytokine detection can distinguish nuclear and cytoplasmic localization [104,105]. Most of the cytokines are localized in the cytoplasm of the producing cell before secretion. However, for some cytokines, such as IL-1 family members IL-1α [106], IL-33 and IL-37 [107,108,109], nuclear localization has been detected. Nuclear localisation of IL-1α is mostly observed in resting cells, where IL-1α is probably acting as a transcription factor regulating cell growth and differentiation [100,109]. Similarly, nuclear localisation of a potent pro-inflammatory cytokine TNFα was detected. The TNFα release from stimulated cells could be blocked by leptomycin B, a specific inhibitor of nuclear protein export [110].

#### 2.2.3. Electrochemiluminescence Immunoassays

Electrochemiluminescence (ECL) is an electrochemical reaction converting electrical energy to emission of light. ECL can be used for sensitive detection of biomolecules (reviewed in [111,112,113]). The first ECL immunoassay has been developed in nineties [114], utilizing ruthenium complexes for electrochemiluminescence reaction [115]. Currently, ECL is widely used in routine and custom immunoassays in clinical, preclinical and research testing. The research ECL immunoassay platforms are commercially available from Meso Scale Diagnostics (MSD) Company (Rockville, MD, USA). Their Multi-Array technology allows simultaneous quantification of one to ten analytes using a convenient format of 96 or 384-well plates. In the bottom of each well, carbon electrodes are placed, each pre-coated with one or several anti-cytokine capture antibodies. After incubation with sample, the detection antibodies, conjugated to an electrochemiluminescent label (sulfo-tag), are added. The sulfo-tags emit light when electricity is applied on the plate electrodes and light intensity is measured to quantify analytes in the sample. The Multi-Arrays ensure low background, as only labels near the electrode surface are detected. The arrays provide high sensitivity, broad dynamic range of quantification and similar results of absolute quantification, comparable to bead-based cytometric assays [116,117,118]. Kits for detection of hundreds of analytes are available with additional possibility to build custom assays.

ECL immunoassays, using both clinical or research platforms, find broad application in cancer research to detect tumour antigens (e.g., CEA, CA-125, PSA) and have also been applied to study cytokines associated with the disease [119,120,121,122].

#### 2.2.4. Antibody Arrays

In the past 10 years, popularity and availability of multiplex immunoassays, that enable quantification of up to tens (theoretically of hundreds) of analytes in a single measurement, have grown rapidly. The reasons arise mainly from the fact that single cytokine is often insufficient as a true disease biomarker. As molecules and cells act in complicated networks and regulation of multiple entities leads to the observed effect, a more global view requiring multiplex platforms is needed to understand true disease biology [123]. Simultaneous measurement of multiple analytes saves time, costs and, importantly, sample requirements. Antibody arrays are mostly used in form of bead-based arrays (such as Luminex assays, Austin, TX, USA) or planar arrays (antibody arrays and antibody microarrays) [123,124].

##### Bead-Based Arrays

Bead based multiplex assays belong to the most used formats of multiplex cytokine assays, due to their high accuracy, sensitivity, reproducibility, broad dynamic range of quantification, high throughput and capability to quantify multiple analytes using as low as 25 to 50 μL of the sample. Such arrays have been built on the principle of sandwich ELISA, with the capture antibodies immobilized on microbeads. The microbeads possess internal fluorescence label that is unique for each measured analyte. The detection antibodies are mostly biotinylated and a streptavidin-phycoerytrhin conjugate is used for cytokine detection and quantification. Cytokine measurement is performed in a flow cytometer or dedicated Luminex instrument based on 2 measured signals. In the typical Luminex settings, the red laser (classification laser) excites the bead internal dye to identify each microsphere particle (analyte). At the same time, the cytokine amount is quantified from fluorescence intensity of phycoerythrin, which has been excited by a green laser (detection laser). 

Two types of microbeads may be used in the assays, polystyren or magnetic beads. Polystyren beads were historically the first developed for Luminex assays. However, their incubation and washing using filter 96-well plates may be accompanied by leaking and clogging problems, leading to reduced accuracy and precision [125]. Such problems have been overcome by development of magnetic beads, with fast and highly effective washing performed by hand-held magnet or automated magnetic plate washer.

Using Luminex assays, theoretically up to 100 analytes can be measured simultaneously. However, to avoid cross reactivity of antibodies and incorrect results, practical level of multiplexing reaches around 30 analytes in one panel. Combination of panels leads to hundreds to thousand detectable analytes. Currently, kits for human, mouse, rat, canine, monkey and porcine samples are available to cover various model organisms, with permanently growing number of covered analytes and species. Moreover, custom assays can be built by antibody coupling to magnetic beads and Luminex Corp. provides support for their customers in custom assay development. 

**Figure 3:**
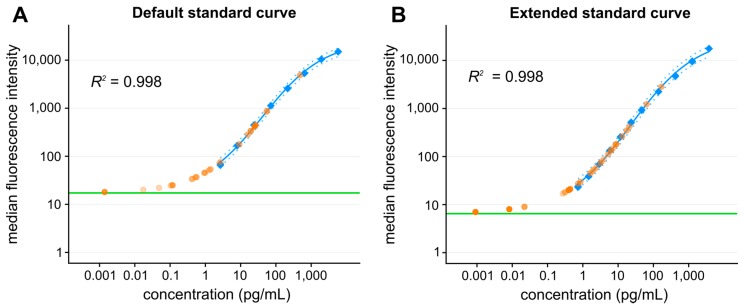
Optimization of standard curve to detect low amounts of IFNα by Luminex array. (**A**) Typical calibration curve covers 3–4 LOGs of cytokine concentration; (**B**) for some cytokines, additional data points at the lower end of the curve enable quantification of low cytokine concentration in samples and expansion of quantification over 4 orders of magnitude in cytokine concentration. Standard curves are calculated by drLumi package [132] in R statistical environment, full line shows standard curve fit to calibration standards (blue diamonds), dotted lines show prediction interval. Data points of real samples of experimental pig model body fluids are shown as orange circles. Three circles with the lowest IFNα concentration represent healthy control untreated animals.

Detection range of bead-based arrays spans over 3–4 orders of magnitude with the sensitivity reaching low picogram/mL concentrations. As in other immunoassays, good pipetting technique is critical to ensure high precision and accurate results. Using precise reversed pipetting and optimized dilution of standards, the lower limit of detection may be expanded below 1 pg/mL for some cytokines (Figure 3) [126]. Correct quantification at the lower end of the calibration curve is often crucial for setting a baseline of the control samples, e.g., in analysis of healthy control human samples or *in vitro* cultured unstimulated cells and non-immune cells, where the cytokine production is mostly low. In such cases, concentration of conditioned cell culture medium prior analysis may seem to be advantageous to increase cytokine concentrations. However, the sample concentration (e.g., by 3 kDa MWCO centrifugation filters) is not bringing the expected benefits. In our experiments, concentration of culture medium samples to 10% of its original volume led to only 1–5 time increase of the cytokines compared to original unconcentrated sample. Cytokine molecule degradation, adhesion to the filter, protein aggregation/precipitation or other factors may participate in the protein loss. Opposite situation occurs in plasma or serum samples, where increasing sample concentration is practically impossible due to already high protein content. Interestingly, from our experience, 2-fold diluted serum or plasma provide better cytokine concentration readings (e.g., lower variation of sample replicates) than use of undiluted samples. Sample dilution may lead to reduction of sample viscosity ensuring better availability of epitopes to antibodies. Reduction of matrix effect in diluted samples was observed also by Rosenberg-Hasson et al. [127].

Bead-based arrays find broad applications in cancer research, including melanoma research. Immunotherapeutic predictive or prognostic values of serum cytokines were tested in melanoma patients treated by adjuvant interferon [128] and ipilimumab [69]. Blood plasma cytokines have been analysed in patients with unresectable in-transit extremity melanoma, treated by isolated limb infusion with melphalan [129]. In uveal melanoma, bead-based arrays have been used to monitor response of PBMCs cells to stimulation by differentiation and tumour antigens [130] and to detect cytokines secreted into vitreous humour [131]. At the cellular level, Luminex assay was used to map cross-talk between fibroblasts and epithelial cells [41].

##### Planar Antibody Arrays

Antibody arrays are widely recognized as a reliable and robust methodology for mining multiplex data from complex proteomes with high sensitivity, high specificity and high throughput [123]. The first antibody arrays have been developed by Chang in 1983 by spotting antibodies in 10 × 10 and 20 × 20 grids on 1 cm^2^ area of glass cover slips [133]. Current arrays are mostly based on sandwich or direct label layout (Figure 2). Sandwich arrays use antibody pairs that have to be validated and checked for cross-reactivity to every other antigen/antibody in the array. From that reasons, practical sizes of the arrays mostly cover between 10 and 80 analytes. By combination of several arrays, detection and quantification of up to 1000 secreted human proteins, such as cytokines, chemokines, adipokines, growth factors, proteases, soluble receptors and other proteins, in human samples can be achieved [134]. In research on animals, up to 200 mouse, 67 rat, 50 porcine, 30 bovine, canine and rabbit, 20 ovine and 10 equine, feline, Rhesus Monkey, chicken and dolphin cytokines can be currently quantitatively measured by combination of antibody arrays (data based on on-line catalogues of major producers Raybiotech, Abcam and RnD).

**Figure 4:**
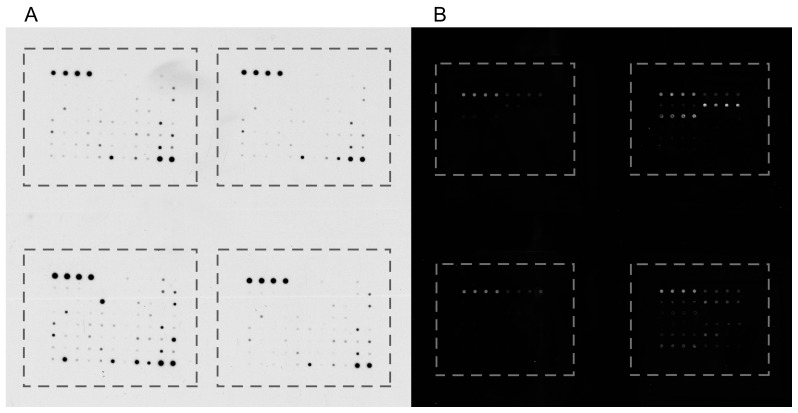
Planar antibody arrays for detection of secreted cytokines. (**A**) Membrane array with chemiluminescent detection; (**B**) glass array with fluorescent detection. Positions of individual subarrays (4 individual samples) are highlighted by dashed line.

Antibodies are printed mostly on nitrocellulose membrane or glass slide as a solid support (Figure 4). Membrane arrays are usually semi-quantitative (enable fold-change readings) with chemiluminescent or fluorescent detection and are easily processed similarly as western blot membranes without the need of specialized laboratory equipment. Glass slide arrays accommodate denser antibody prints and are mostly quantitative with fluorescent detection. Scanning of fluorescent arrays requires array scanner but is commonly offered as a payed service. For easy handling, glass slide arrays mostly consist of 2 × 8 subarrays, arranged in the format of 2 columns of the 96-well plate, enabling analysis of up to 16 samples or calibration standards on one slide. Four slides can be adopted into a frame resembling 96-well plate, enabling automation. Antibodies are spotted in several replicates for variability evaluation and proper quantification.

Bead-based and planar antibody arrays have been utilized or are potentially applicable to a wide range of cancer studies, including search for diagnostic and prognostic biomarkers, evaluation of antitumour treatment efficacy, understanding of the biology underlining tumourigenesis and tumour progression [123,135,136], including mapping the role of tumour microenvironment and inter-cellular cross-talk [137].

### 2.3. Emerging Techniques for Ultrasensitive Detection of Secreted Cytokines

Current technological improvements led to development of ultrasensitive techniques that significantly enhance the detection of analytes at low concentration in pg/mL to fg/mL range [138,139,140]. Beside cytokine analysis, the ultrasensitive techniques are designed for detection of biomarkers present in the blood plasma/serum at very low levels to facilitate diagnostics of early stages of diseases, such as in neurodegenerative [140], cardiovascular diseases [141], inflammation or cancer [142]. Ultrasensitive methods are proposed to improve sensitivity, selectivity, simplicity and minimize the sample matrix effects together with minimizing the sample volumes. In addition to traditional colorimetric, chemiluminescent and fluorescent detection, the emerging technologies rely on electrochemical, optical [143], mechanical or surface plasmon resonance [144] biosensing, reviewed in [74,142,145,146,147]. Developments in nanotechnologies enable construction of microfluidic devices and biochips [74]. Moreover, concentration of an analyte to minimal volumes using femtoliter-sized wells, optical fibres, microfluidics compartments, or nanodroplets enables readout at the level of single events [148,149]. Combination of microfluidics and nanotechnologies with a variety of ultrasensitive reporters has a potential to reach unprecedented sensitivities approaching 1000 protein molecules per microliter, i.e., the aM–fM range [142,150]. However, most of the emerging techniques utilize purified proteins and simple sample matrices. The robustness of such techniques in the context of complex biological samples still remains to be demonstrated before their potential application in biological research and clinical practice [142].

Selected more or less established ultrasensitive technologies are mentioned in the following paragraphs. Due to the large variety of emerging technologies for cytokine biosensing, it is out of the scope of this review to provide global information on all of them. Several reviews have been published in the last 3 years, where the more detailed information on biosensing can be found [59,74,143,144,145,146].

#### 2.3.1. Single Molecule Counting (Singulex)

Single Molecule Counting (SMC) technology, originally developed by Singulex Inc. (Alameda, CA, USA), combines bead-based immunoassay with Single Molecule Counting detection [151,152]. Classical 96-well plate format bead-based immunoassay is used to form sandwich complexes (magnetic bead coated by capture antibody—antigen—fluorescently labelled detection antibody). Then, the sandwich complex is disrupted and eluted fluorescently-labelled detection antibodies are analysed using proprietary digital SMC technology. SMC counts single molecule fluorescent signals (“flashes”) using laser confocal microscope digital counter. SMC assay kits offer detection of cytokines in human samples with sensitivity in sub-pg/mL levels. Custom assays for various analytes can be built. Adaptation of the SMC technology employing 3 different lasers enabled multiplex (3-plex) detection of IL-4, IL-6 and IL-10 levels in blood plasma of healthy donors with single pg/mL sensitivity [153].

#### 2.3.2. Single Molecule Array (Simoa)

Single Molecule Array (Simoa), developed by Quanterix Corp. (Lexington, MA, USA), is a digital ELISA that reaches astonishing sensitivity up to sub fg/mL concentration [138]. Simoa is based on classical sandwich immunoassay on magnetic beads followed by digital detection. Antigen is captured by an antibody immobilized on magnetic microbeads and by addition of biotinylated detection antibody and streptavidin-labelled enzyme, an immunocomplex is formed. Beads are then loaded into a microwell array consisting of 50,000 wells (46 fL wells, each can accommodate only one bead) and sealed in the presence of fluorogenic substrate. Due to extremely small reaction volume, the concentration of fluorescent product easily reaches a detectable range even if only one enzyme molecule is present in the well [154]. Very low analyte concentrations, i.e., zero or one antigen on each bead, can be directly determined from number of fluorescent wells and total number of beads in the array by digital readout (0 or 1 signal). In higher analyte concentrations, direct quantification from fluorescence intensity (analogue readout) is applied. Simoa is open for multiplexing by using beads labelled by distinct fluorescent dyes [155] and currently up to 10 analytes can be simultaneously measured. Multiplex Simoa for simultaneous detection of 6 cytokines showed 0.01–0.03 pg/mL limit of detection [156]. In another study, Simoa assays for 10 cytokines in human serum samples have shown limit of detection between 0.001–0.217 pg/mL [154]. Kits for various analytes are available from Quanterix Corp.

#### 2.3.3. Immuno-PCR

ImmunoPCR, originally described in 1992 [157], takes advantage of high specificity of immunoassays and high sensitivity of polymerase chain reaction (PCR). Polymerase enzyme allows for an exponential signal amplification compared to the linear relation between substrate and product in conventional ELISA and provides up to 1000× higher sensitivity than common ELISA [158,159,160].

ImmunoPCR in sandwich format, identically to sandwich ELISA, is performed in 96-well plate coated by the capture antibody. Unlike to enzyme-antibody conjugates used for detection in ELISA assay, the immunoPCR utilizes detection antibody covalently conjugated to DNA and detected by quantitative PCR. Commercial ready-to-use covalent conjugates (Imperacer^®^, Chimera Biotec, Dortmund, Germany) or kits for their production are now available [159]. Detailed protocol for immunoPCR assay development for IL-6 in plasma was described by [161]. Applications of immuno-PCR for the detection of early stage cancer were currently reviewed in [162,163].

Although the merits of immuno-PCR are obvious (e.g., ultra-sensitivity, good reproducibility and universality), its main limitation is a high background and the need of extensive washing steps [160]. Proximity ligation assay (PLA) and Proximity extension assay (PEA) have been developed recently to reduce nonspecific binding and shorten processing time of immunoPCR.

#### 2.3.4. Proximity Ligation Assay

Proximity ligation assay (PLA) was originally described by Fredriksson et al. [164], who used a DNA aptamer with affinity to platelet-derived growth factor (PDGF) protein to quantify PGDF. Homodimer PDGF-BB can accommodate two aptamer molecules, each of them having extensions for primer binding and additional extension to be joined by ligation upon hybridization to a common connector oligonucleotide. Real-time detection of PCR products could detect as low as 10^−20^ molar protein concentrations [164].

In current proximity ligation assays, antibodies binding pairwise to adjacent epitopes of target proteins are used. In case of direct PLA, such antibodies are biotinylated and combined with oligonucleotides covalently attached to streptavidin at either their 5′- or 3′-ends. In indirect PLA, unmodified primary antibodies risen in 2 different species are used, which are detected with secondary antibodies conjugated to the DNA strand [165].

Kits and reagents are now commercialized, including Duolink^®^ (Merck, Darmstadt, Germany), TaqMan^®^ Protein Assay (Thermo Fisher Scientific, Waltham, MA USA), ProQuantum High-Sensitivity Immunoassays (Thermo Fisher Scientific) or Proximity Ligation Assay (Abnova, Taipei, Taiwan). The applications of PLA enable also studies of protein interactions, phosphorylations or *in situ* subcellular localisation.

#### 2.3.5. Proximity Extension Assay

Proximity Extension Assay (PEA) is an alternative to PLA. The main difference between the two techniques is that the ligation event of PLA is replaced by a DNA polymerisation step in PEA, which minimizes the background noise and improves assay sensitivity [165]. PEA assays use matched antibody pairs linked to either 3′ or 5′ ends of unique single-stranded DNA sequences. The other (unoccupied) ends of ssDNA are complementary, allowing for pair-wise annealing with the other oligonucleotide and extension by a DNA polymerase. Each oligonucleotide contains a primer-binding site. Thus, only in case of specific binding of both antibodies to one protein and complementary ssDNA sequence hybridization, the PCR product is formed and quantified by qPCR. Such PEA has been commercialized by Olink (Uppsala, Sweden). Unlike conventional immunoassays, where multiplexing degree is limited by unspecific binding (cross reactivity) of antibodies, in PEA only matched DNAs are amplified, which enables high specificity and high degree of multiplexing. The Proseek Multiplex panels (Olink) enable analysis of 92 protein biomarkers across 96 samples simultaneously, using 1 μL of sample.

#### 2.3.6. ImmunoMagnetic Reduction Assay

Magnetic susceptibility reduction as a bioanalytical technique was described in 2006 [166], where biotinylated magnetic nanoparticles were used to detect avidin. Current immuno magnetic reduction (IMR) assays are using capture antibodies immobilized on magnetic nanoparticles. Such nanoparticles are homogeneously dispersed in a solution and oscillate by application of external multiple alternating current (AC) magnetic fields (i.e., are susceptible to the magnetic field). Upon addition of the analysed sample, nanoparticles become heavier due to the capture of analyte molecules by antibodies. This results in reduced response of nanoparticles to the magnetic field. Degree of reduction of AC magnetic susceptibility corresponds to the amount of the target molecules present in the sample. Highly sensitive magnetometer detectors enable to detect proteins at pg/mL concentrations [167].

The main advantage of the IMR assay is that it is simple technique that does not need washing steps to remove unbound reagents. Moreover, the assay is highly sensitive and quantitative. For example, concentration ranges 1–50,000 pg/mL and 0.3 fg/mL–300 pg/mL of amyloid-β1-42 and α-synuclein, respectively, could be covered [147,167]. Unlike sandwich immunoassays, the IMR assay uses just one antibody (“capture” antibody). The assay specificity is ensured by oscillatory movements of nanoparticles, where weak non-specific antigen-antibody interactions are disrupted due to centrifugal forces evoked on nanoparticles by application of magnetic fields.

The IMR assay is currently aimed at identification of early stages of diseases, such as neurodegenerative diseases [147,167,168], cancer [169,170,171] and viral infections [172]. In cancer research, assay kits for VEGF [169] and carcinoembryonic antigen [170] are available.

Despite sensitive and elegant cytokine quantification by immunotechniques, the absolute cytokine concentrations should be interpreted with caution. An extensive inter-assay comparison of 9 emerging or well-established platforms for cytokine quantification was performed by Yeung et al. to evaluate 4 selected cytokine (IL-6, TNFα, IL-17a, IL-2) levels [173]. Despite using the same reference set of human serum samples, different immunoassays yielded different quantitative results. With a primary focus on assay sensitivity and accuracy, Simoa (Quanterix) and Erenna (Singulex) platforms showed the best performance, followed by V-plex (MSD) and Ella (ProteinSimple; an automated ELISA using microfluidic cartridge) assays [173]. Similar comparison of 14 immunoassays (ELISA, bead-based arrays and MSD) with absorbance, chemiluminescence, electrochemiluminescence and fluorescence detection of IL-1β and IL-6 showed significant inter-laboratory and inter-assay variations [174].

### 2.4. Single-Cell Analyses

Immunoassays are frequently aimed at measuring cytokine production by large numbers of cells, such as cytokines released into the tumour tissue, to body fluids (e.g., blood) or cytokines released by cells cultured *in vitro*. Thus, the cytokine producing cell populations are mostly heterogeneous and only the average response of cells is measured. Analysis of rare subsets of cells (like antigen-specific T-cells or B-cells) by common methods is challenging, if not impossible, as such cells contribute only a minor component of the total measurement [175]. Thus, techniques to detect cytokine production by single cells have been developed.

#### 2.4.1. ELISpot

Enzyme-linked ImmunoSpot (ELISpot) assay has been originally developed for detection of antibody producing cells [176]. Later, ELISpot was adapted to enumerating of activated T-cells secreting IFNγ among human peripheral blood lymphocytes [177]. Since then, ELISpot became popular as an easy and highly quantitative assay for detection of single cell producing cytokine and monitoring cellular response to various stimuli [178].

In this technique, cells are incubated in wells of 96-well plate coated with a capture antibody that captures the secreted cytokine. After the removal of cells, enzyme-conjugated detection antibody is added and cytokine production is visualized by a colour reaction. Position of each cytokine-producing cell is visualized as a spot. Number and size of the spots is detected by ELISpot reader and Spot forming cells (SFC) are counted [179,180].

ELISpot is adaptable not only to the evaluation of a variety of T-cell functions but also to B-cells and innate immune cells. In cancer research, ELISpot is often employed for evaluation of T-cell response to cancer therapy in phase I and II trials. ELISpot might be a useful biomarker assay to predict cancer vaccine efficacy [178,181].

#### 2.4.2. Flow Cytometry

**Figure 5:**
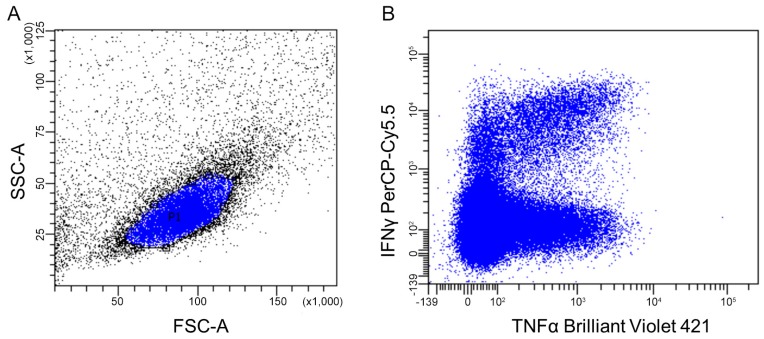
Application of flow cytometry to detect intracellular cytokines in porcine lymphocytes. (**A**) Activated cells with lymphoid scatter characteristics (highlighted in blue) from Melanoma–bearing Libechov Minipigs; (**B**) expression of IFNγ and TNFα in these cells.

The first device, which had all the fundamental elements of a flow cytometer, was built by Louis Kamentsky in 1965 [182]. Interestingly, the first cell sorter was introduced in the same year [183]. Flow cytometry is based on single-cell analysis, which allows to show the frequency of cytokine-expressing cells together with characterization of producing cell population using staining of intracellular cytokines together with cell markers [184,185] (Figure 5). In the intracellular cytokine staining, cells are activated to produce cytokines and at the same time treated by substances that prevent cytokine secretion (such as Brefeldin A or monensin [186]). Subsequently, the cells are fixed and permeabilized to allow binding of the specific anti-cytokine antibodies [187,188,189]. Flow cytometry allows multiparameter analysis and the number of analysed parameters depends on the cell population, cytokines of interest and the sophistication of the cytometer. However, not all cytokines can be detected using a single protocol and demanding optimization of staining may be required to detect dim, low-frequency, or newly characterized cytokines [184]. Moreover, the presence of a cytokine within cells does not necessarily mean that it would be released from cells or exhibit biological effects *in vivo* [190].

The most frequently studied cytokine producing cells are T-lymphocytes [190] but also other cell populations, including basophils [191], neutrophils [192,193], NK cells [194], monocytes [192] and mast cells [195] are investigated. In melanoma, flow cytometry was applied to study mechanisms of effects of immunotherapies, such as a PD-1 protein blockade by monoclonal antibodies pembrolizumab or nivolumab [196,197,198], CTLA-4 blockade with ipilimumab [198,199,200,201] or high-dose IL-2 therapy [202,203]. Intracellular cytokine staining can be used to monitor tumour antigen-specific T-cell functions [204,205,206] in the search for anti-tumour competent cytotoxic T-cells.

#### 2.4.3. Mass Cytometry

In 2009, Bandura et al. developed new approach combining flow cytometry and mass spectrometry—mass cytometry (also called CyTOF) [207]. This approach allows real time quantitative single cell analysis of theoretically up to 3000 cells per second and simultaneous measurement of 60 distinct markers (proteins or other biomolecules) in one cell. As in classical flow cytometry, cells are stained by specific antibodies but these antibodies are conjugated with element tags (stable isotopes of metals) instead of fluorescent dyes. Analysis of cells is performed by inductively coupled plasma time-of-flight mass spectrometer (ICPTOF-MS). The main advantages of this technique are high resolution and sensitivity together with high number of markers for simultaneous measurement with no need for compensation of emission spectral overlaps in comparison to fluorescence-based flow cytometry [207]. However, this technique has also several drawbacks—a lack of forward and side light scatter information, impossibility of cell sorting implementation (because cells are vaporized during analysis) and challenging quality control and statistical analyses of results [207,208]. Study of a human hematopoietic continuum stimulated by cytokines proved that results from mass cytometry are comparable and provide similar information value as fluorescence flow cytometry [209].

Although mass cytometry is a relatively new technique, it is well-established and broadly accepted by scientific community and protocols for intracellular cytokine staining for mass cytometry analysis are similar to protocols for classical flow cytometry analyses [208,210]. However, only several studies characterized cytokine production in immune cells using mass cytometry. Fisher et al. noticed dysregulated production of cytokines in myelofibrosis, when they monitored 12 selected cytokines, e.g., IFNγ, IL-6, MIP1β and TNFα [211]. O’Gorman et al. analysed expression of 16 cytokines (IL-1α, IL-1β, IL-1RA, IL-2, IL-4, IL-6, IL-8, IL-12p40, IL-17A, Perforin, GM-CSF, IFNα, IFNγ, MCP1, MIP1β and TNFα) in monocytes in patients with systemic lupus erythematosus [210]. Developments in multiplex mass cytometry for assessment of T-cell antigen specificity, applicable in search for targets of anti-cancer therapies, are discussed in [212].

#### 2.4.4. Single Cell Arrays

Although the ELISPOT assay and flow cytometry are widely accepted in the preclinical testing, they rely on analysis of cells cultured in bulks, where the cells are under influence of paracrine factors produced by other cells. Response of individual cells (e.g., T-cell stimulation by cancer antigen) can be monitored by currently developed microtools for single secretome analysis. Accommodation of single cells into separated compartments can be achieved in valved microfluidic chambers (similar to microfluidic chips used to single-cell RNA sequencing) or in an array of subnanoliter wells [175].

Initial single cell array for secreted cytokines employed 81,400 microwells (~0.1 nL each) moulded on a poly(dimethylsiloxane) slab. The array was loaded with PBMCs cells (~1 cell per well) and then inverted onto a glass slide coated with a specific capture anti-IL-6 or anti-IFNγ antibody. After incubation, appropriate detection antibodies conjugated to fluorescent dyes were added and the fluorescence was recorded by microarray scanner [213]. In the following study, anti-IFNγ capture antibodies were incorporated into a hydrogel that was used for coating of a glass slide. Microwells of 20 μm diameter were formed by photolithography on top of the antibody-containing hydrogel layer and filled with cells. Such array was used to determine IFN-γ secretion by individual stimulated T-lymphocytes [214].

Simultaneous detection of multiple cytokines was achieved by further improvement of the single cell arrays. Han et al. used an array of 125 picolitre volume wells to accommodate single cells. Such an array was covered by a cover-slip, coated by mixture of 3 capture antibodies. Mixture of detection antibodies conjugated to 3 distinct fluorescent labels and a microarray scanner have been used to simultaneously determine levels of IFN-γ, IL-2 and TNFα in a secretome of stimulated T-cells [215]. In another study, high-density antibody barcode array chip together with subnanolitre microchamber array were used to determine secretion of 14 cytokines. The assay performance was documented for both cell lines (human A549 and U937 cell lines) as well as primary cultures isolated from human tumours (glioblastoma, meningioma) [216]. Further combination of spatial and spectral encoding led to co-detection of 42 immune effector proteins in single cells [217].

In focus on melanoma, Ma et al. developed a microfluidic chip for quantification of dozen effector molecules secreted from tumour antigen–specific cytotoxic T-lymphocytes that were actively responding to melanoma and compared with healthy donor controls [218]. Two types of tumour antigen-specific cytotoxic T-cell were evaluated: (i) T-cells transgenic for T-cell receptor specific for melanoma-associated antigen recognized by T-cells 1 (MART-1) protein and (ii) *ex vivo*–expanded tyrosinase-specific T-cells. A microfluidic chip with 3 nanolitre volume microcompartments was used to detect secretion of 10^4^ individual cells. The cytokine production was detected by dense bar code antibody array—set of 12 capture antibodies immobilized in form of bars across each chip microcompartment. After incubation with cells, chip chambers have been flushed with biotinylated detection antibody and streptavidin-phycoerythrin was used for target quantification. Although the individual T-cells of each type were phenotypically similar, functional heterogeneity in active tumour antigen–specific CTLs was observed among single cells [218]. The heterogeneity among phenotypically similar cytokine producing cells was documented not only for T-lymphocytes but also for macrophages [217,219] and NK-cells [220]. The single-cell studies demonstrate that only very low percentage of immune cells gets activated upon stimulation.

### 2.5. Other Techniques for Cytokine Detection

Immunoassays are powerful techniques to quantify cytokines but they may provide only limited information on the cytokine activity. As the immunoassays may suffer from detection of biologically inactive cytokines (e.g., IL-6 bound to its soluble receptor [221]) or cytokine fragments, or may be influenced by matrix effects, antibody unspecific binding or cross-reactivity, bioassays can be applied to detect biological activity of cytokines. Bioassays are using cells (and, mostly historically, also experimental animals) to monitor cytokine effects. The monitored target cell response may include induction of proliferation, differentiation or growth inhibition, chemotaxis, phagocytosis, cytotoxicity/apoptosis, induction of antiviral or antimicrobial activity, or up-regulation of expression of intracellular, surface membrane or secreted proteins [98,222]. Despite their unique power to measure biological activity of cytokines, bioassays, like all biological systems, are inherently variable and also laborious. Thus, bioassays are increasingly replaced by immunoassays, even at the cost of loss of information on the cytokine activity [222].

Many other techniques are applied beyond proteomics to detect cytokines. Analysis of cytokine gene expression is robust, fast and easy technique applicable even to very limited sample amounts. However, the relation between mRNA level and activity of the secreted protein is difficult to estimate, mainly due to regulations on posttranscriptional [223] as well as posttranslational level (secretory pathway, storage prior release, activating proteolytic cleavage, binding of secreted cytokine to neutralizing molecules, etc.). Nevertheless, transcriptomic analysis in combination with other techniques contributes to comprehensive view on cytokine regulation in biologic systems.

Immunohistochemistry and immunocytochemistry represent additional inseparable part of cancer and melanoma research. Such techniques enable visualization of antigen expression patterns and immune response across the tissue of cells. Immunofluorescence staining of intracellular cytokines as described in Flow cytometry section can be also analysed using regular or confocal fluorescence microscope. This technique does not allow sensitive and precise quantification but can provide additional information on morphology and identity of the cytokine producing cells.

## 3. Conclusions

Melanoma may serve as a model for tumour immuno-oncology. Melanoma spontaneous regression accompanied by tumour infiltration by T-lymphocytes suggest the active role of immune system in tumour management. Knowledge of immune system response to melanoma represents one of the key prerequisites for development of immunotherapies to manage cancers. Cytokines are the main regulatory molecules in the immune system. Significant progress in proteomics and other technologies enable monitoring of cytokine release with very high sensitivity. Simultaneous analysis of several molecules by multiplex techniques provides more global view of cytokine regulatory networks. Single-cell technologies open new ways of research towards functions of individual cells.

Further developments in multiplex and ultrasensitive techniques are expected in the next few years, including developments in antibodies and aptamers for cytokine molecule capturing or developments in sensing. However, careful testing should be applied to correctly quantify cytokines in real biological and patient samples by emerging analytical techniques. Inter-individual variability and inter-assay variability should be taken into account when summarizing results from various studies.

Knowledge gained from cytokine analysis is expected to facilitate identification of targets and monitoring of efficiency of melanoma immunotherapies.

## Abbreviations

bFGF	basic fibroblast growth factor
CAFs	cancer-associated fibroblasts
CCL	C-C motif chemokine
CTLA-4	T-lymphocyte-associated antigen 4
CXCL1	chemokine (C-X-C motif) ligand 1 (GROα)
DDA	data dependent acquisition
DIA	data independent acquisition
DIADI	direct immunoaffinity desorption/ionization
ECL	electrochemiluminescence
ELISpot	enzyme-linked immunospot assay
EVs	extracellular vesicles
G-CSF	granulocyte-colony stimulating factor
GDF15	growth/differentiation factor 15
GM-CSF	granulocyte-macrophage colony-stimulating factor
GROα	GRO1 oncogene
HGF	hepatocyte growth factor
HPLC	high-performance liquid chromatography
IFN	interferon
IGF	insulin-like growth factor
IL	interleukin
IL-10	interferon gamma-induced protein 10
IL1-ra	interleukin-1 receptor antagonist
IMR	immunomagnetic reduction
KGF	keratinocyte growth factor
MALDI	matrix-assisted laser desorption ionisation
MART-1	melanoma-associated antigen recognized by T cells 1
MCP-1	monocyte chemoattractant protein 1
M-CSF	macrophage colony-stimulating factor
MIF	macrophage migration inhibitory factor
MIP-1β	macrophage inflammatory protein-1β
MRM	multiple reaction monitoring
MS	mass spectrometry
MS/MS	tandem mass spectrometry
NK	natural killer
NRG-1	neuregulin-1
PBMCs	peripheral blood mononuclear cells
PD-1	programmed cell-death protein 1
PDGF	platelet-derived growth factor
PD-ligand	ligand of PD-1 receptor
PEA	proximity extension assay
PLA	proximity ligation assay
SCF	stem cell factor
Simoa	single molecule array
SISCAPA	stable isotope standards and capture by anti-peptide antibodies
SMC	single molecule counting
SRM	selected reaction monitoring
SWATH-MS	sequential windowed acquisition of all theoretical mass spectra
TAMs	tumour-associated macrophages
TARC	thymus and activation regulated chemokine (CCL17)
TGFβ	transforming growth factor-β
TNFα	tumour necrosis factor-α
Tregs	regulatory T-cells
VEGF-A	vascular endothelial growth factor A

## References

[1] Ferlay J., Steliarova-Foucher E., Lortet-Tieulent J., Rosso S., Coebergh J.W.W., Comber H., Forman D., Bray F. (2013). Cancer incidence and mortality patterns in Europe: Estimates for 40 countries in 2012. Eur. J. Cancer.

[2] Guy G.P., Thomas C.C., Thompson T., Watson M., Massetti G.M., Richardson L.C. (2015). Vital signs: Melanoma incidence and mortality trends and projections—United States, 1982–2030. Morb. Mortal. Wkly. Rep..

[3] Dunki-Jacobs E.M., Callender G.G., McMasters K.M. (2013). Current management of melanoma. Curr. Probl. Surg..

[4] Lo J.A., Fisher D.E. (2014). The melanoma revolution: From UV carcinogenesis to a new era in therapeutics. Science.

[5] Ali Z., Yousaf N., Larkin J. (2013). Melanoma epidemiology, biology and prognosis. EJC Suppl. EJC Off. J. EORTC Eur. Organ. Res. Treat. Cancer Al.

[6] Gilchrest B.A., Eller M.S., Geller A.C., Yaar M. (1999). The pathogenesis of melanoma induced by ultraviolet radiation. N. Engl. J. Med..

[7] Lea C.S., Scotto J.A., Buffler P.A., Fine J., Barnhill R.L., Berwick M. (2007). Ambient UVB and melanoma risk in the United States: A case-control analysis. Ann. Epidemiol..

[8] Rivers J.K. (1996). Melanoma. Lancet.

[9] Beaumont K.A., Mohana-Kumaran N., Haass N.K. (2013). Modeling Melanoma *In Vitro* and *In Vivo*. Healthcare.

[10] Kuzu O.F., Nguyen F.D., Noory M.A., Sharma A. (2015). Current State of Animal (Mouse) Modeling in Melanoma Research. Cancer Growth Metastasis.

[11] Van der Weyden L., Patton E.E., Wood G.A., Foote A.K., Brenn T., Arends M.J., Adams D.J. (2016). Cross-species models of human melanoma. J. Pathol..

[12] Bourneuf E. (2017). The MeLiM Minipig: An Original Spontaneous Model to Explore Cutaneous Melanoma Genetic Basis. Front. Genet..

[13] Cole W.H., Everson T.C. (1956). Spontaneous regression of cancer: Preliminary report. Ann. Surg..

[14] High W.A., Stewart D., Wilbers C.R.H., Cockerell C.J., Hoang M.P., Fitzpatrick J.E. (2005). Completely regressed primary cutaneous malignant melanoma with nodal and/or visceral metastases: A report of 5 cases and assessment of the literature and diagnostic criteria. J. Am. Acad. Dermatol..

[15] Blessing K., McLaren K.M. (1992). Histological regression in primary cutaneous melanoma: Recognition, prevalence and significance. Histopathology.

[16] Haanen J.B.A.G. (2013). Immunotherapy of melanoma. EJC Suppl. EJC Off. J. EORTC Eur. Organ. Res. Treat. Cancer Al.

[17] Kalialis L.V., Drzewiecki K.T., Klyver H. (2009). Spontaneous regression of metastases from melanoma: Review of the literature. Melanoma Res..

[18] Aung P.P., Nagarajan P., Prieto V.G. (2017). Regression in primary cutaneous melanoma: Etiopathogenesis and clinical significance. Lab. Investig. J. Tech. Methods Pathol..

[19] Maio M. (2012). Melanoma as a model tumour for immuno-oncology. Ann. Oncol. Off. J. Eur. Soc. Med. Oncol..

[20] Martín J.M., Pinazo I., Mateo J.F., Escandell I., Jordá E., Monteagudo C. (2014). Assessment of regression in successive primary melanomas. Actas Dermosifiliogr..

[21] Creagan E.T., Ahmann D.L., Green S.J., Long H.J., Frytak S., O’Fallon J.R., Itri L.M. (1984). Phase II study of low-dose recombinant leukocyte A interferon in disseminated malignant melanoma. J. Clin. Oncol. Off. J. Am. Soc. Clin. Oncol..

[22] Robinson W.A., Mughal T.I., Thomas M.R., Johnson M., Spiegel R.J. (1986). Treatment of metastatic malignant melanoma with recombinant interferon alpha 2. Immunobiology.

[23] Rosenberg S.A., Lotze M.T., Muul L.M., Chang A.E., Avis F.P., Leitman S., Linehan W.M., Robertson C.N., Lee R.E., Rubin J.T. (1987). A progress report on the treatment of 157 patients with advanced cancer using lymphokine-activated killer cells and interleukin-2 or high-dose interleukin-2 alone. N. Engl. J. Med..

[24] Dutcher J.P., Creekmore S., Weiss G.R., Margolin K., Markowitz A.B., Roper M., Parkinson D., Ciobanu N., Fisher R.I., Boldt D.H. (1989). A phase II study of interleukin-2 and lymphokine-activated killer cells in patients with metastatic malignant melanoma. J. Clin. Oncol. Off. J. Am. Soc. Clin. Oncol..

[25] Maker A.V., Phan G.Q., Attia P., Yang J.C., Sherry R.M., Topalian S.L., Kammula U.S., Royal R.E., Haworth L.R., Levy C. (2005). Tumor regression and autoimmunity in patients treated with cytotoxic T lymphocyte-associated antigen 4 blockade and interleukin 2: A phase I/II study. Ann. Surg. Oncol..

[26] Aris M., Mordoh J., Barrio M.M. (2017). Immunomodulatory Monoclonal Antibodies in Combined Immunotherapy Trials for Cutaneous Melanoma. Front. Immunol..

[27] Ryu S., Youn C., Moon A.R., Howland A., Armstrong C.A., Song P.I. (2017). Therapeutic Inhibitors against Mutated BRAF and MEK for the Treatment of Metastatic Melanoma. Chonnam Med. J..

[28] Dudley M.E., Wunderlich J.R., Robbins P.F., Yang J.C., Hwu P., Schwartzentruber D.J., Topalian S.L., Sherry R., Restifo N.P., Hubicki A.M. (2002). Cancer regression and autoimmunity in patients after clonal repopulation with antitumor lymphocytes. Science.

[29] Atkins M.B., Hsu J., Lee S., Cohen G.I., Flaherty L.E., Sosman J.A., Sondak V.K., Kirkwood J.M., Eastern Cooperative Oncology Group (2008). Phase III trial comparing concurrent biochemotherapy with cisplatin, vinblastine, dacarbazine, interleukin-2 and interferon alfa-2b with cisplatin, vinblastine and dacarbazine alone in patients with metastatic malignant melanoma (E3695): A trial coordinated by the Eastern Cooperative Oncology Group. J. Clin. Oncol. Off. J. Am. Soc. Clin. Oncol..

[30] Seung S.K., Curti B.D., Crittenden M., Walker E., Coffey T., Siebert J.C., Miller W., Payne R., Glenn L., Bageac A. (2012). Phase 1 study of stereotactic body radiotherapy and interleukin-2—Tumor and immunological responses. Sci. Transl. Med..

[31] Sosman J.A., Carrillo C., Urba W.J., Flaherty L., Atkins M.B., Clark J.I., Dutcher J., Margolin K.A., Mier J., Gollob J. (2008). Three phase II cytokine working group trials of gp100 (210M) peptide plus high-dose interleukin-2 in patients with HLA-A2-positive advanced melanoma. J. Clin. Oncol. Off. J. Am. Soc. Clin. Oncol..

[32] Schwartzentruber D.J., Lawson D.H., Richards J.M., Conry R.M., Miller D.M., Treisman J., Gailani F., Riley L., Conlon K., Pockaj B. (2011). gp100 peptide vaccine and interleukin-2 in patients with advanced melanoma. N. Engl. J. Med..

[33] Luke J.J., Flaherty K.T., Ribas A., Long G.V. (2017). Targeted agents and immunotherapies: Optimizing outcomes in melanoma. Nat. Rev. Clin. Oncol..

[34] Dvořánková B., Szabo P., Kodet O., Strnad H., Kolář M., Lacina L., Krejčí E., Naňka O., Šedo A., Smetana K. (2017). Intercellular crosstalk in human malignant melanoma. Protoplasma.

[35] Lacina L., Kodet O., Dvořánková B., Szabo P., Smetana K. (2018). Ecology of melanoma cell. Histol. Histopathol..

[36] Lacina L., Plzak J., Kodet O., Szabo P., Chovanec M., Dvorankova B., Smetana K. (2015). Cancer Microenvironment: What Can We Learn from the Stem Cell Niche. Int. J. Mol. Sci..

[37] Paulitschke V., Kunstfeld R., Mohr T., Slany A., Micksche M., Drach J., Zielinski C., Pehamberger H., Gerner C. (2009). Entering a new era of rational biomarker discovery for early detection of melanoma metastases: Secretome analysis of associated stroma cells. J. Proteome Res..

[38] D’Orazio J., Jarrett S., Amaro-Ortiz A., Scott T. (2013). UV radiation and the skin. Int. J. Mol. Sci..

[39] Kodet O., Lacina L., Krejčí E., Dvořánková B., Grim M., Štork J., Kodetová D., Vlček Č., Šáchová J., Kolář M. (2015). Melanoma cells influence the differentiation pattern of human epidermal keratinocytes. Mol. Cancer.

[40] Wang Y., Viennet C., Robin S., Berthon J.-Y., He L., Humbert P. (2017). Precise role of dermal fibroblasts on melanocyte pigmentation. J. Dermatol. Sci..

[41] Kolář M., Szabo P., Dvořánková B., Lacina L., Gabius H.-J., Strnad H., Sáchová J., Vlček C., Plzák J., Chovanec M. (2012). Upregulation of IL-6, IL-8 and CXCL-1 production in dermal fibroblasts by normal/malignant epithelial cells *in vitro*: Immunohistochemical and transcriptomic analyses. Biol. Cell.

[42] Jobe N.P., Rösel D., Dvořánková B., Kodet O., Lacina L., Mateu R., Smetana K., Brábek J. (2016). Simultaneous blocking of IL-6 and IL-8 is sufficient to fully inhibit CAF-induced human melanoma cell invasiveness. Histochem. Cell Biol..

[43] Hoejberg L., Bastholt L., Schmidt H. (2012). Interleukin-6 and melanoma. Melanoma Res..

[44] Singh S., Singh A.P., Sharma B., Owen L.B., Singh R.K. (2010). CXCL8 and its cognate receptors in melanoma progression and metastasis. Future Oncol..

[45] Kučera J., Dvořánková B., Smetana K., Szabo P., Kodet O. (2015). Fibroblasts isolated from the malignant melanoma influence phenotype of normal human keratinocytes. J. Appl. Biomed..

[46] Gasser S., Lim L.H.K., Cheung F.S.G. (2017). The role of the tumour microenvironment in immunotherapy. Endocr. Relat. Cancer.

[47] Fløe A., Løppke C., Hilberg O., Wejse C., Brix L., Jacobsen K. (2017). Development of an epitope panel for consistent identification of antigen-specific T-cells in humans. Immunology.

[48] Zikich D., Schachter J., Besser M.J. (2016). Predictors of tumor-infiltrating lymphocyte efficacy in melanoma. Immunotherapy.

[49] Ouyang Z., Wu H., Li L., Luo Y., Li X., Huang G. (2016). Regulatory T cells in the immunotherapy of melanoma. Tumour Biol. J. Int. Soc. Oncodev. Biol. Med..

[50] Fujimura T., Kakizaki A., Furudate S., Kambayashi Y., Aiba S. (2016). Tumor-associated macrophages in skin: How to treat their heterogeneity and plasticity. J. Dermatol. Sci..

[51] Mignogna C., Scali E., Camastra C., Presta I., Zeppa P., Barni T., Donato G., Bottoni U., Di Vito A. (2017). Innate immunity in cutaneous melanoma. Clin. Exp. Dermatol..

[52] Tarazona R., Duran E., Solana R. (2015). Natural Killer Cell Recognition of Melanoma: New Clues for a More Effective Immunotherapy. Front. Immunol..

[53] Saadeh D., Kurban M., Abbas O. (2016). Plasmacytoid dendritic cell role in cutaneous malignancies. J. Dermatol. Sci..

[54] Chiaruttini G., Mele S., Opzoomer J., Crescioli S., Ilieva K.M., Lacy K.E., Karagiannis S.N. (2017). B cells and the humoral response in melanoma: The overlooked players of the tumor microenvironment. Oncoimmunology.

[55] Weidle U.H., Birzele F., Kollmorgen G., Rüger R. (2017). The Multiple Roles of Exosomes in Metastasis. Cancer Genom. Proteom..

[56] O’Loghlen A. (2018). Role for extracellular vesicles in the tumour microenvironment. Philos. Trans. R. Soc. Lond. B Biol. Sci..

[57] Romano G., Kwong L.N. (2017). miRNAs, Melanoma and Microenvironment: An Intricate Network. Int. J. Mol. Sci..

[58] Ratnikov B.I., Scott D.A., Osterman A.L., Smith J.W., Ronai Z.A. (2017). Metabolic rewiring in melanoma. Oncogene.

[59] Stenken J.A., Poschenrieder A.J. (2015). Bioanalytical chemistry of cytokines—A review. Anal. Chim. Acta.

[60] Yao M., Brummer G., Acevedo D., Cheng N. (2016). Cytokine Regulation of Metastasis and Tumorigenicity. Adv. Cancer Res..

[61] Atretkhany K.-S.N., Drutskaya M.S., Nedospasov S.A., Grivennikov S.I., Kuprash D.V. (2016). Chemokines, cytokines and exosomes help tumors to shape inflammatory microenvironment. Pharmacol. Ther..

[62] Herraiz C., Jiménez-Cervantes C., Sánchez-Laorden B., García-Borrón J.C. (2017). Functional interplay between secreted ligands and receptors in melanoma. Semin. Cell Dev. Biol..

[63] Liu Q., Li A., Tian Y., Wu J.D., Liu Y., Li T., Chen Y., Han X., Wu K. (2016). The CXCL8-CXCR1/2 pathways in cancer. Cytokine Growth Factor Rev..

[64] Sanmamed M.F., Carranza-Rua O., Alfaro C., Oñate C., Martín-Algarra S., Perez G., Landazuri S.F., Gonzalez A., Gross S., Rodriguez I. (2014). Serum interleukin-8 reflects tumor burden and treatment response across malignancies of multiple tissue origins. Clin. Cancer Res. Off. J. Am. Assoc. Cancer Res..

[65] Alegre E., Sammamed M., Fernandez-Landazuri S., Zubiri L., Gonzalez A. (2015). Circulating Biomarkers in Malignant Melanoma. Advances in Clinical Chemistry.

[66] Filitis D.C., Rauh J., Mahalingam M. (2015). The HGF-cMET signaling pathway in conferring stromal-induced BRAF-inhibitor resistance in melanoma. Melanoma Res..

[67] Matsumoto K., Umitsu M., De Silva D.M., Roy A., Bottaro D.P. (2017). Hepatocyte growth factor/MET in cancer progression and biomarker discovery. Cancer Sci..

[68] Lok E., Chung A.S., Swanson K.D., Wong E.T. (2014). Melanoma brain metastasis globally reconfigures chemokine and cytokine profiles in patient cerebrospinal fluid. Melanoma Res..

[69] Najjar Y.G., Ding F., Lin Y., VanderWeele R., Butterfield L.H., Tarhini A.A. (2017). Melanoma antigen-specific effector T cell cytokine secretion patterns in patients treated with ipilimumab. J. Transl. Med..

[70] Xu D.H., Zhu Z., Xiao H., Wakefield M.R., Bai Q., Nicholl M.B., Ding V.A., Fang Y. (2017). Unveil the mysterious mask of cytokine-based immunotherapy for melanoma. Cancer Lett..

[71] Jiang T., Zhou C., Ren S. (2016). Role of IL-2 in cancer immunotherapy. Oncoimmunology.

[72] Ives N.J., Suciu S., Eggermont A.M.M., Kirkwood J., Lorigan P., Markovic S.N., Garbe C., Wheatley K., International Melanoma Meta-Analysis Collaborative Group (IMMCG) (2017). Adjuvant interferon-α for the treatment of high-risk melanoma: An individual patient data meta-analysis. Eur. J. Cancer.

[73] Hoeller C., Michielin O., Ascierto P.A., Szabo Z., Blank C.U. (2016). Systematic review of the use of granulocyte-macrophage colony-stimulating factor in patients with advanced melanoma. Cancer Immunol. Immunother..

[74] Liu G., Qi M., Hutchinson M.R., Yang G., Goldys E.M. (2016). Recent advances in cytokine detection by immunosensing. Biosens. Bioelectron..

[75] Kulbe H., Chakravarty P., Leinster D.A., Charles K.A., Kwong J., Thompson R.G., Coward J.I., Schioppa T., Robinson S.C., Gallagher W.M. (2012). A dynamic inflammatory cytokine network in the human ovarian cancer microenvironment. Cancer Res..

[76] Nilsson T., Mann M., Aebersold R., Yates J.R., Bairoch A., Bergeron J.J.M. (2010). Mass spectrometry in high-throughput proteomics: Ready for the big time. Nat. Methods.

[77] Anderson N.L., Anderson N.G. (2002). The human plasma proteome: History, character and diagnostic prospects. Mol. Cell. Proteom..

[78] Rocco M., Malorni L., Cozzolino R., Palmieri G., Rozzo C., Manca A., Parente A., Chambery A. (2011). Proteomic profiling of human melanoma metastatic cell line secretomes. J. Proteome Res..

[79] Alečković M., Wei Y., LeRoy G., Sidoli S., Liu D.D., Garcia B.A., Kang Y. (2017). Identification of Nidogen 1 as a lung metastasis protein through secretome analysis. Genes Dev..

[80] Boyle G.M., Pedley J., Martyn A.C., Banducci K.J., Strutton G.M., Brown D.A., Breit S.N., Parsons P.G. (2009). Macrophage inhibitory cytokine-1 is overexpressed in malignant melanoma and is associated with tumorigenicity. J. Investig. Dermatol..

[81] Rosenberger G., Koh C.C., Guo T., Röst H.L., Kouvonen P., Collins B.C., Heusel M., Liu Y., Caron E., Vichalkovski A. (2014). A repository of assays to quantify 10,000 human proteins by SWATH-MS. Sci. Data.

[82] Collins B.C., Hunter C.L., Liu Y., Schilling B., Rosenberger G., Bader S.L., Chan D.W., Gibson B.W., Gingras A.-C., Held J.M. (2017). Multi-laboratory assessment of reproducibility, qualitative and quantitative performance of SWATH-mass spectrometry. Nat. Commun..

[83] Anjo S.I., Santa C., Manadas B. (2017). SWATH-MS as a tool for biomarker discovery: From basic research to clinical applications. Proteomics.

[84] Lin Q., Lim H.S.R., Lin H.L., Tan H.T., Lim T.K., Cheong W.K., Cheah P.Y., Tang C.L., Chow P.K.H., Chung M.C.M. (2015). Analysis of colorectal cancer glyco-secretome identifies laminin β-1 (LAMB1) as a potential serological biomarker for colorectal cancer. Proteomics.

[85] Manfredi M., Martinotti S., Gosetti F., Ranzato E., Marengo E. (2016). The secretome signature of malignant mesothelioma cell lines. J. Proteom..

[86] Addona T.A., Abbatiello S.E., Schilling B., Skates S.J., Mani D.R., Bunk D.M., Spiegelman C.H., Zimmerman L.J., Ham A.-J.L., Keshishian H. (2009). Multi-site assessment of the precision and reproducibility of multiple reaction monitoring-based measurements of proteins in plasma. Nat. Biotechnol..

[87] Percy A.J., Chambers A.G., Yang J., Hardie D.B., Borchers C.H. (2014). Advances in multiplexed MRM-based protein biomarker quantitation toward clinical utility. Biochim. Biophys. Acta.

[88] Parker C.E., Borchers C.H. (2014). Mass spectrometry based biomarker discovery, verification and validation—Quality assurance and control of protein biomarker assays. Mol. Oncol..

[89] Bredehöft M., Schänzer W., Thevis M. (2008). Quantification of human insulin-like growth factor-1 and qualitative detection of its analogues in plasma using liquid chromatography/electrospray ionisation tandem mass spectrometry. Rapid Commun. Mass Spectrom..

[90] Anderson N.L., Anderson N.G., Haines L.R., Hardie D.B., Olafson R.W., Pearson T.W. (2004). Mass spectrometric quantitation of peptides and proteins using Stable Isotope Standards and Capture by Anti-Peptide Antibodies (SISCAPA). J. Proteome Res..

[91] Kuhn E., Whiteaker J.R., Mani D.R., Jackson A.M., Zhao L., Pope M.E., Smith D., Rivera K.D., Anderson N.L., Skates S.J. (2012). Interlaboratory evaluation of automated, multiplexed peptide immunoaffinity enrichment coupled to multiple reaction monitoring mass spectrometry for quantifying proteins in plasma. Mol. Cell. Proteom..

[92] Sherma N.D., Borges C.R., Trenchevska O., Jarvis J.W., Rehder D.S., Oran P.E., Nelson R.W., Nedelkov D. (2014). Mass Spectrometric Immunoassay for the qualitative and quantitative analysis of the cytokine Macrophage Migration Inhibitory Factor (MIF). Proteome Sci..

[93] Pompach P., Benada O., Rosůlek M., Darebná P., Hausner J., Růžička V., Volný M., Novák P. (2016). Protein Chips Compatible with MALDI Mass Spectrometry Prepared by Ambient Ion Landing. Anal. Chem..

[94] Pompach P., Nováková J., Kavan D., Benada O., Růžička V., Volný M., Novák P. (2016). Planar Functionalized Surfaces for Direct Immunoaffinity Desorption/Ionization Mass Spectrometry. Clin. Chem..

[95] Engvall E., Jonsson K., Perlmann P. (1971). Enzyme-linked immunosorbent assay. II. Quantitative assay of protein antigen, immunoglobulin G, by means of enzyme-labelled antigen and antibody-coated tubes. Biochim. Biophys. Acta.

[96] Van Weemen B.K., Schuurs A.H.W.M. (1971). Immunoassay using antigen-enzyme conjugates. FEBS Lett..

[97] Shah K., Maghsoudlou P. (2016). Enzyme-linked immunosorbent assay (ELISA): The basics. Br. J. Hosp. Med..

[98] Whiteside T.L. (2002). Cytokine assays. BioTechniques.

[99] Towbin H., Staehelin T., Gordon J. (1979). Electrophoretic transfer of proteins from polyacrylamide gels to nitrocellulose sheets: Procedure and some applications. Proc. Natl. Acad. Sci. USA.

[100] Apte R.N., Dotan S., Elkabets M., White M.R., Reich E., Carmi Y., Song X., Dvozkin T., Krelin Y., Voronov E. (2006). The involvement of IL-1 in tumorigenesis, tumor invasiveness, metastasis and tumor-host interactions. Cancer Metastasis Rev..

[101] Schneider K.S., Thomas C.J., Groß O. (2013). Inflammasome activation and inhibition in primary murine bone marrow-derived cells and assays for IL-1α, IL-1β and caspase-1. Methods Mol. Biol..

[102] Guey B., Petrilli V. (2016). Assessing Caspase-1 Activation. Methods Mol. Biol..

[103] Logan P., Burnier J., Burnier M.N. (2013). Vascular endothelial growth factor expression and inhibition in uveal melanoma cell lines. Ecancermedicalscience.

[104] Gatla H.R., Singha B., Persaud V., Vancurova I. (2014). Evaluating cytoplasmic and nuclear levels of inflammatory cytokines in cancer cells by western blotting. Methods Mol. Biol..

[105] Miskolci V., Hodgson L., Cox D., Vancurova I. (2014). Western analysis of intracellular interleukin-8 in human mononuclear leukocytes. Methods Mol. Biol..

[106] Wessendorf J.H., Garfinkel S., Zhan X., Brown S., Maciag T. (1993). Identification of a nuclear localization sequence within the structure of the human interleukin-1 alpha precursor. J. Biol. Chem..

[107] Boraschi D., Lucchesi D., Hainzl S., Leitner M., Maier E., Mangelberger D., Oostingh G.J., Pfaller T., Pixner C., Posselt G. (2011). IL-37: A new anti-inflammatory cytokine of the IL-1 family. Eur. Cytokine Netw..

[108] Ross R., Grimmel J., Goedicke S., Möbus A.M., Bulau A.-M., Bufler P., Ali S., Martin M.U. (2013). Analysis of nuclear localization of interleukin-1 family cytokines by flow cytometry. J. Immunol. Methods.

[109] Bertheloot D., Latz E. (2017). HMGB1, IL-1α, IL-33 and S100 proteins: Dual-function alarmins. Cell. Mol. Immunol..

[110] Miskolci V., Ghosh C.C., Rollins J., Romero C., Vu H.-Y., Robinson S., Davidson D., Vancurova I. (2006). TNFalpha release from peripheral blood leukocytes depends on a CRM1-mediated nuclear export. Biochem. Biophys. Res. Commun..

[111] Richter M.M. (2004). Electrochemiluminescence (ECL). Chem. Rev..

[112] Rhyne P.W., Wong O.T., Zhang Y.J., Weiner R.S. (2009). Electrochemiluminescence in bioanalysis. Bioanalysis.

[113] Wei H., Wang E. (2011). Electrochemiluminescence of tris(2,2′-bipyridyl)ruthenium and its applications in bioanalysis: A review. Lumin. J. Biol. Chem. Lumin..

[114] Obenauer-Kutner L.J., Jacobs S.J., Kolz K., Tobias L.M., Bordens R.W. (1997). A highly sensitive electrochemiluminescence immunoassay for interferon alfa-2b in human serum. J. Immunol. Methods.

[115] Hercules D.M., Lytle F.E. (1966). Chemiluminescence from Reduction Reactions. J. Am. Chem. Soc..

[116] Chowdhury F., Williams A., Johnson P. (2009). Validation and comparison of two multiplex technologies, Luminex and Mesoscale Discovery, for human cytokine profiling. J. Immunol. Methods.

[117] Fu Q., Zhu J., Van Eyk J.E. (2010). Comparison of multiplex immunoassay platforms. Clin. Chem..

[118] Dabitao D., Margolick J.B., Lopez J., Bream J.H. (2011). Multiplex measurement of proinflammatory cytokines in human serum: Comparison of the Meso Scale Discovery electrochemiluminescence assay and the Cytometric Bead Array. J. Immunol. Methods.

[119] Ryan B.M., Pine S.R., Chaturvedi A.K., Caporaso N., Harris C.C. (2014). A combined prognostic serum interleukin-8 and interleukin-6 classifier for stage 1 lung cancer in the prostate, lung, colorectal and ovarian cancer screening trial. J. Thorac. Oncol. Off. Publ. Int. Assoc. Study Lung Cancer.

[120] Block M.S., Maurer M.J., Goergen K., Kalli K.R., Erskine C.L., Behrens M.D., Oberg A.L., Knutson K.L. (2015). Plasma immune analytes in patients with epithelial ovarian cancer. Cytokine.

[121] Pan Y.W., Zhou Z.G., Wang M., Dong J.Q., Du K.P., Li S., Liu Y.L., Lv P.J., Gao J.B. (2016). Combination of IL-6, IL-10 and MCP-1 with traditional serum tumor markers in lung cancer diagnosis and prognosis. Genet. Mol. Res..

[122] Shimizu Y., Furuya H., Bryant Greenwood P., Chan O., Dai Y., Thornquist M.D., Goodison S., Rosser C.J. (2016). A multiplex immunoassay for the non-invasive detection of bladder cancer. J. Transl. Med..

[123] Wilson J.J., Burgess R., Mao Y.-Q., Luo S., Tang H., Jones V.S., Weisheng B., Huang R.-Y., Chen X., Huang R.-P. (2015). Antibody arrays in biomarker discovery. Adv. Clin. Chem..

[124] Valekova I., Skalnikova H.K., Jarkovska K., Motlik J., Kovarova H. (2015). Multiplex immunoassays for quantification of cytokines, growth factors and other proteins in stem cell communication. Methods Mol. Biol..

[125] Faresjö M. (2014). A useful guide for analysis of immune markers by fluorochrome (Luminex) technique. Methods Mol. Biol..

[126] Valekova I., Jarkovska K., Kotrcova E., Bucci J., Ellederova Z., Juhas S., Motlik J., Gadher S.J., Kovarova H. (2016). Revelation of the IFNα, IL-10, IL-8 and IL-1β as promising biomarkers reflecting immuno-pathological mechanisms in porcine Huntington’s disease model. J. Neuroimmunol..

[127] Rosenberg-Hasson Y., Hansmann L., Liedtke M., Herschmann I., Maecker H.T. (2014). Effects of serum and plasma matrices on multiplex immunoassays. Immunol. Res..

[128] Tarhini A.A., Lin Y., Zahoor H., Shuai Y., Butterfield L.H., Ringquist S., Gogas H., Sander C., Lee S., Agarwala S.S. (2015). Pro-Inflammatory Cytokines Predict Relapse-Free Survival after One Month of Interferon-α but Not Observation in Intermediate Risk Melanoma Patients. PLoS ONE.

[129] Shetty G., Beasley G.M., Sparks S., Barfield M., Masoud M., Mosca P.J., Pruitt S.K., Salama A.K.S., Chan C., Tyler D.S. (2013). Plasma cytokine analysis in patients with advanced extremity melanoma undergoing isolated limb infusion. Ann. Surg. Oncol..

[130] Triozzi P.L., Aldrich W., Crabb J.W., Singh A.D. (2015). Spontaneous cellular and humoral tumor antigen responses in patients with uveal melanoma. Melanoma Res..

[131] Ly L.V., Bronkhorst I.H.G., van Beelen E., Vrolijk J., Taylor A.W., Versluis M., Luyten G.P.M., Jager M.J. (2010). Inflammatory cytokines in eyes with uveal melanoma and relation with macrophage infiltration. Investig. Ophthalmol. Vis. Sci..

[132] Sanz H., Aponte J.J., Harezlak J., Dong Y., Ayestaran A., Nhabomba A., Mpina M., Maurin O.R., Díez-Padrisa N., Aguilar R. (2017). drLumi: An open-source package to manage data, calibrate and conduct quality control of multiplex bead-based immunoassays data analysis. PLoS ONE.

[133] Chang T.W. (1983). Binding of cells to matrixes of distinct antibodies coated on solid surface. J. Immunol. Methods.

[134] Antibody Arrays for Protein Detection. https://www.raybiotech.com/antibody-array.

[135] Kopf E., Zharhary D. (2007). Antibody arrays—An emerging tool in cancer proteomics. Int. J. Biochem. Cell Biol..

[136] Sanchez-Carbayo M. (2010). Antibody array-based technologies for cancer protein profiling and functional proteomic analyses using serum and tissue specimens. Tumour Biol. J. Int. Soc. Oncodev. Biol. Med..

[137] Gál P., Varinská L., Fáber L., Novák Š., Szabo P., Mitrengová P., Mirossay A., Mučaji P., Smetana K. (2017). How Signaling Molecules Regulate Tumor Microenvironment: Parallels to Wound Repair. Molecules.

[138] Rissin D.M., Kan C.W., Campbell T.G., Howes S.C., Fournier D.R., Song L., Piech T., Patel P.P., Chang L., Rivnak A.J. (2010). Single-molecule enzyme-linked immunosorbent assay detects serum proteins at subfemtomolar concentrations. Nat. Biotechnol..

[139] Fischer S.K., Joyce A., Spengler M., Yang T.-Y., Zhuang Y., Fjording M.S., Mikulskis A. (2015). Emerging technologies to increase ligand binding assay sensitivity. AAPS J..

[140] Andreasson U., Blennow K., Zetterberg H. (2016). Update on ultrasensitive technologies to facilitate research on blood biomarkers for central nervous system disorders. Alzheimers Dement. Amst. Neth..

[141] Smith J.G., Gerszten R.E. (2017). Emerging Affinity-Based Proteomic Technologies for Large-Scale Plasma Profiling in Cardiovascular Disease. Circulation.

[142] Simon S., Ezan E. (2017). Ultrasensitive bioanalysis: Current status and future trends. Bioanalysis.

[143] Singh M., Truong J., Reeves W.B., Hahm J.-I. (2017). Emerging Cytokine Biosensors with Optical Detection Modalities and Nanomaterial-Enabled Signal Enhancement. Sensors.

[144] Rodríguez-Frade J.M., Martínez-Muñoz L., Villares R., Cascio G., Lucas P., Gomariz R.P., Mellado M. (2016). Chemokine Detection Using Receptors Immobilized on an SPR Sensor Surface. Methods Enzymol..

[145] Zhou Q., Son K., Liu Y., Revzin A. (2015). Biosensors for Cell Analysis. Annu. Rev. Biomed. Eng..

[146] Chen P., Huang N.-T., Chung M.-T., Cornell T.T., Kurabayashi K. (2015). Label-free cytokine micro- and nano-biosensing towards personalized medicine of systemic inflammatory disorders. Adv. Drug Deliv. Rev..

[147] Yang X., Tang Y., Alt R.R., Xie X., Li F. (2016). Emerging techniques for ultrasensitive protein analysis. Analyst.

[148] Cretich M., Daaboul G.G., Sola L., Ünlü M.S., Chiari M. (2015). Digital detection of biomarkers assisted by nanoparticles: Application to diagnostics. Trends Biotechnol..

[149] Zhang Y., Noji H. (2017). Digital Bioassays: Theory, Applications and Perspectives. Anal. Chem..

[150] Ahn S., Zhang P., Yu H., Lee S., Kang S.H. (2016). Ultrasensitive Detection of α-Fetoprotein by Total Internal Reflection Scattering-Based Super-Resolution Microscopy for Superlocalization of Nano-Immunoplasmonics. Anal. Chem..

[151] Wu A.H.B., Fukushima N., Puskas R., Todd J., Goix P. (2006). Development and preliminary clinical validation of a high sensitivity assay for cardiac troponin using a capillary flow (single molecule) fluorescence detector. Clin. Chem..

[152] Todd J., Freese B., Lu A., Held D., Morey J., Livingston R., Goix P. (2007). Ultrasensitive flow-based immunoassays using single-molecule counting. Clin. Chem..

[153] Gilbert M., Livingston R., Felberg J., Bishop J.J. (2016). Multiplex single molecule counting technology used to generate interleukin 4, interleukin 6 and interleukin 10 reference limits. Anal. Biochem..

[154] Wu D., Milutinovic M.D., Walt D.R. (2015). Single molecule array (Simoa) assay with optimal antibody pairs for cytokine detection in human serum samples. Analyst.

[155] Rissin D.M., Kan C.W., Song L., Rivnak A.J., Fishburn M.W., Shao Q., Piech T., Ferrell E.P., Meyer R.E., Campbell T.G. (2013). Multiplexed single molecule immunoassays. Lab. Chip.

[156] Rivnak A.J., Rissin D.M., Kan C.W., Song L., Fishburn M.W., Piech T., Campbell T.G., DuPont D.R., Gardel M., Sullivan S. (2015). A fully-automated, six-plex single molecule immunoassay for measuring cytokines in blood. J. Immunol. Methods.

[157] Sano T., Smith C.L., Cantor C.R. (1992). Immuno-PCR: Very sensitive antigen detection by means of specific antibody-DNA conjugates. Science.

[158] Adler M., Spengler M. (2009). Novel Strategies and Tools for Enhanced Sensitivity in Routine Biomolecule Analytics. Curr. Pharm. Anal..

[159] Ryazantsev D.Y., Voronina D.V., Zavriev S.K. (2016). Immuno-PCR: Achievements and Perspectives. Biochem. Biokhimiia.

[160] Chang L., Li J., Wang L. (2016). Immuno-PCR: An ultrasensitive immunoassay for biomolecular detection. Anal. Chim. Acta.

[161] Niemeyer C.M., Adler M., Wacker R. (2007). Detecting antigens by quantitative immuno-PCR. Nat. Protoc..

[162] Khan A.H., Sadroddiny E. (2016). Application of immuno-PCR for the detection of early stage cancer. Mol. Cell. Probes.

[163] Assumpção A.L.F.V., da Silva R.C. (2016). Immuno-PCR in cancer and non-cancer related diseases: A review. Vet. Q..

[164] Fredriksson S., Gullberg M., Jarvius J., Olsson C., Pietras K., Gústafsdóttir S.M., Ostman A., Landegren U. (2002). Protein detection using proximity-dependent DNA ligation assays. Nat. Biotechnol..

[165] Greenwood C., Ruff D., Kirvell S., Johnson G., Dhillon H.S., Bustin S.A. (2015). Proximity assays for sensitive quantification of proteins. Biomol. Detect. Quantif..

[166] Hong C.-Y., Wu C.C., Chiu Y.C., Yang S.Y., Horng H.E., Yang H.C. (2006). Magnetic susceptibility reduction method for magnetically labeled immunoassay. Appl. Phys. Lett..

[167] Yang S.-Y., Chiu M.-J., Chen T.-F., Horng H.-E. (2017). Detection of Plasma Biomarkers Using Immunomagnetic Reduction: A Promising Method for the Early Diagnosis of Alzheimer’s Disease. Neurol. Ther..

[168] Lue L.-F., Sabbagh M.N., Chiu M.-J., Jing N., Snyder N.L., Schmitz C., Guerra A., Belden C.M., Chen T.-F., Yang C.-C. (2017). Plasma Levels of Aβ42 and Tau Identified Probable Alzheimer’s Dementia: Findings in Two Cohorts. Front. Aging Neurosci..

[169] Huang K.W., Yang S.Y., Yu C.Y., Chieh J.J., Yang C.-C., Horng H.-E., Hong C.-Y., Yang H.-C., Wu C.-C. (2011). Exploration of the relationship between the tumor burden and the concentration of vascular endothelial growth factor in liver-cancer-bearing animals using immunomagnetic reduction assay. J. Biomed. Nanotechnol..

[170] Yang C.-C., Yang S.-Y., Ho C.-S., Chang J.-F., Liu B.-H., Huang K.-W. (2014). Development of antibody functionalized magnetic nanoparticles for the immunoassay of carcinoembryonic antigen: A feasibility study for clinical use. J. Nanobiotechnol..

[171] Chieh J.-J., Huang K.W., Chuang C.P., Wei W.C., Dong J.J., Lee Y.Y. (2016). Immunomagnetic Reduction Assay on Des-Gamma-Carboxy Prothrombin for Screening of Hepatocellular Carcinoma. IEEE Trans. Biomed. Eng..

[172] Product-IMR Reagent | MagQu. http://www.magqu.com/product/IMR%20Reagent?shs_term_node_tid_depth=39.

[173] Yeung D., Ciotti S., Purushothama S., Gharakhani E., Kuesters G., Schlain B., Shen C., Donaldson D., Mikulskis A. (2016). Evaluation of highly sensitive immunoassay technologies for quantitative measurements of sub-pg/mL levels of cytokines in human serum. J. Immunol. Methods.

[174] Fichorova R.N., Richardson-Harman N., Alfano M., Belec L., Carbonneil C., Chen S., Cosentino L., Curtis K., Dezzutti C.S., Donoval B. (2008). Biological and technical variables affecting immunoassay recovery of cytokines from human serum and simulated vaginal fluid: A multicenter study. Anal. Chem..

[175] Chattopadhyay P.K., Gierahn T.M., Roederer M., Love J.C. (2014). Single-cell technologies for monitoring immune systems. Nat. Immunol..

[176] Czerkinsky C.C., Nilsson L.A., Nygren H., Ouchterlony O., Tarkowski A. (1983). A solid-phase enzyme-linked immunospot (ELISPOT) assay for enumeration of specific antibody-secreting cells. J. Immunol. Methods.

[177] Czerkinsky C., Andersson G., Ekre H.P., Nilsson L.A., Klareskog L., Ouchterlony O. (1988). Reverse ELISPOT assay for clonal analysis of cytokine production. I. Enumeration of gamma-interferon-secreting cells. J. Immunol. Methods.

[178] Slota M., Lim J.-B., Dang Y., Disis M.L. (2011). ELISpot for measuring human immune responses to vaccines. Expert Rev. Vaccines.

[179] Hauer A.C., Bajaj-Elliott M. (2001). Elispot Technique for Assaying Interleukins. Interleukin Protocols.

[180] Faresjö M. (2014). The challenge of measuring elusive immune markers by enzyme-linked immuno-spot (ELISPOT) technique. Methods Mol. Biol..

[181] Morse M.A., Osada T., Hobeika A., Patel S., Lyerly H.K. (2013). Biomarkers and correlative endpoints for immunotherapy trials. Am. Soc. Clin. Oncol. Educ. Book Am. Soc. Clin. Oncol. Meet..

[182] Kamentsky L.A., Melamed M.R., Derman H. (1965). Spectrophotometer: New instrument for ultrarapid cell analysis. Science.

[183] Fulwyler M.J. (1965). Electronic separation of biological cells by volume. Science.

[184] Yin Y., Mitson-Salazar A., Prussin C. (2015). Detection of Intracellular Cytokines by Flow Cytometry. Curr. Protoc. Immunol..

[185] Freer G. (2014). Intracellular staining and detection of cytokines by fluorescence-activated flow cytometry. Methods Mol. Biol..

[186] Schuerwegh A.J., Stevens W.J., Bridts C.H., De Clerck L.S. (2001). Evaluation of monensin and brefeldin A for flow cytometric determination of interleukin-1 beta, interleukin-6 and tumor necrosis factor-alpha in monocytes. Cytometry.

[187] Sander B., Andersson J., Andersson U. (1991). Assessment of cytokines by immunofluorescence and the paraformaldehyde-saponin procedure. Immunol. Rev..

[188] Jung T., Schauer U., Heusser C., Neumann C., Rieger C. (1993). Detection of intracellular cytokines by flow cytometry. J. Immunol. Methods.

[189] Prussin C., Metcalfe D.D. (1995). Detection of intracytoplasmic cytokine using flow cytometry and directly conjugated anti-cytokine antibodies. J. Immunol. Methods.

[190] Foster B., Prussin C., Liu F., Whitmire J.K., Whitton J.L. (2007). Detection of intracellular cytokines by flow cytometry. Curr. Protoc. Immunol..

[191] Mukai K., Gaudenzio N., Gupta S., Vivanco N., Bendall S.C., Maecker H.T., Chinthrajah R.S., Tsai M., Nadeau K.C., Galli S.J. (2017). Assessing basophil activation by using flow cytometry and mass cytometry in blood stored 24 hours before analysis. J. Allergy Clin. Immunol..

[192] Schmidt C.S., Aranda Lopez P., Dopheide J.F., Schmidt F., Theobald M., Schild H., Lauinger-Lörsch E., Nolte F., Radsak M.P. (2016). Phenotypic and functional characterization of neutrophils and monocytes from patients with myelodysplastic syndrome by flow cytometry. Cell. Immunol..

[193] Manfredi A.A., Rovere-Querini P., D’Angelo A., Maugeri N. (2017). Low molecular weight heparins prevent the induction of autophagy of activated neutrophils and the formation of neutrophil extracellular traps. Pharmacol. Res..

[194] Misale M.S., Witek Janusek L., Tell D., Mathews H.L. (2018). Chromatin organization as an indicator of glucocorticoid induced natural killer cell dysfunction. Brain. Behav. Immun..

[195] Yin Y., Bai Y., Olivera A., Desai A., Metcalfe D.D. (2017). An optimized protocol for the generation and functional analysis of human mast cells from CD34(+) enriched cell populations. J. Immunol. Methods.

[196] Daud A.I., Loo K., Pauli M.L., Sanchez-Rodriguez R., Sandoval P.M., Taravati K., Tsai K., Nosrati A., Nardo L., Alvarado M.D. (2016). Tumor immune profiling predicts response to anti-PD-1 therapy in human melanoma. J. Clin. Investig..

[197] Ribas A., Shin D.S., Zaretsky J., Frederiksen J., Cornish A., Avramis E., Seja E., Kivork C., Siebert J., Kaplan-Lefko P. (2016). PD-1 Blockade Expands Intratumoral Memory T Cells. Cancer Immunol. Res..

[198] Tietze J.K., Angelova D., Heppt M.V., Reinholz M., Murphy W.J., Spannagl M., Ruzicka T., Berking C. (2017). The proportion of circulating CD45RO(+)CD8(+) memory T cells is correlated with clinical response in melanoma patients treated with ipilimumab. Eur. J. Cancer.

[199] Kitano S., Tsuji T., Liu C., Hirschhorn-Cymerman D., Kyi C., Mu Z., Allison J.P., Gnjatic S., Yuan J.D., Wolchok J.D. (2013). Enhancement of tumor-reactive cytotoxic CD4+ T cell responses after ipilimumab treatment in four advanced melanoma patients. Cancer Immunol. Res..

[200] De Coaña Y.P., Wolodarski M., Poschke I., Yoshimoto Y., Yang Y., Nyström M., Edbäck U., Brage S.E., Lundqvist A., Masucci G.V. (2017). Ipilimumab treatment decreases monocytic MDSCs and increases CD8 effector memory T cells in long-term survivors with advanced melanoma. Oncotarget.

[201] Wistuba-Hamprecht K., Martens A., Heubach F., Romano E., Geukes Foppen M., Yuan J., Postow M., Wong P., Mallardo D., Schilling B. (2017). Peripheral CD8 effector-memory type 1 T-cells correlate with outcome in ipilimumab-treated stage IV melanoma patients. Eur. J. Cancer.

[202] Diller M.L., Kudchadkar R.R., Delman K.A., Lawson D.H., Ford M.L. (2016). Complete response to high-dose IL-2 and enhanced IFNγ+Th17 :  TREG ratio in a melanoma patient. Melanoma Res..

[203] Diller M.L., Kudchadkar R.R., Delman K.A., Lawson D.H., Ford M.L. (2016). Exogenous IL-2 Induces FoxP3+ Th17 Cells *In Vivo* in Melanoma Patients. J. Immunother..

[204] Zelba H., Weide B., Martens A., Derhovanessian E., Bailur J.K., Kyzirakos C., Pflugfelder A., Eigentler T.K., Di Giacomo A.M., Maio M. (2014). Circulating CD4+ T cells that produce IL4 or IL17 when stimulated by melan-A but not by NY-ESO-1 have negative impacts on survival of patients with stage IV melanoma. Clin. Cancer Res. Off. J. Am. Assoc. Cancer Res..

[205] Zelba H., Weide B., Martens A., Bailur J.K., Garbe C., Pawelec G. (2015). The prognostic impact of specific CD4 T-cell responses is critically dependent on the target antigen in melanoma. Oncoimmunology.

[206] Borchers S., Maβlo C., Müller C.A., Tahedl A., Volkind J., Nowak Y., Umansky V., Esterlechner J., Frank M.H., Ganss C. (2017). Detection of ABCB5 tumour antigen-specific CD8(+) T cells in melanoma patients and implications for immunotherapy. Clin. Exp. Immunol..

[207] Bandura D.R., Baranov V.I., Ornatsky O.I., Antonov A., Kinach R., Lou X., Pavlov S., Vorobiev S., Dick J.E., Tanner S.D. (2009). Mass cytometry: Technique for real time single cell multitarget immunoassay based on inductively coupled plasma time-of-flight mass spectrometry. Anal. Chem..

[208] Cosma A., Nolan G., Gaudilliere B. (2017). Mass cytometry: The time to settle down. Cytom. Part J. Int. Soc. Anal. Cytol..

[209] Bendall S.C., Simonds E.F., Qiu P., Amir E.D., Krutzik P.O., Finck R., Bruggner R.V., Melamed R., Trejo A., Ornatsky O.I. (2011). Single-cell mass cytometry of differential immune and drug responses across a human hematopoietic continuum. Science.

[210] O’Gorman W.E., Kong D.S., Balboni I.M., Rudra P., Bolen C.R., Ghosh D., Davis M.M., Nolan G.P., Hsieh E.W.Y. (2017). Mass cytometry identifies a distinct monocyte cytokine signature shared by clinically heterogeneous pediatric SLE patients. J. Autoimmun..

[211] Fisher D.A.C., Miner C.A., Engle E.K., Brost T.M., Malkova O., Oh S.T. (2016). Mass Cytometry Analysis of Dysregulated Cytokine Production and Intracellular Signaling in Myelofibrosis. Blood.

[212] Newell E.W., Lin W. (2014). High-dimensional analysis of human CD8(+) T cell phenotype, function and antigen specificity. Curr. Top. Microbiol. Immunol..

[213] Bradshaw E.M., Kent S.C., Tripuraneni V., Orban T., Ploegh H.L., Hafler D.A., Love J.C. (2008). Concurrent detection of secreted products from human lymphocytes by microengraving: Cytokines and antigen-reactive antibodies. Clin. Immunol..

[214] Zhu H., Stybayeva G., Silangcruz J., Yan J., Ramanculov E., Dandekar S., George M.D., Revzin A. (2009). Detecting cytokine release from single T-cells. Anal. Chem..

[215] Han Q., Bagheri N., Bradshaw E.M., Hafler D.A., Lauffenburger D.A., Love J.C. (2012). Polyfunctional responses by human T cells result from sequential release of cytokines. Proc. Natl. Acad. Sci. USA.

[216] Lu Y., Chen J.J., Mu L., Xue Q., Wu Y., Wu P.-H., Li J., Vortmeyer A.O., Miller-Jensen K., Wirtz D. (2013). High-throughput secretomic analysis of single cells to assess functional cellular heterogeneity. Anal. Chem..

[217] Lu Y., Xue Q., Eisele M.R., Sulistijo E.S., Brower K., Han L., Amir E.-A.D., Pe’er D., Miller-Jensen K., Fan R. (2015). Highly multiplexed profiling of single-cell effector functions reveals deep functional heterogeneity in response to pathogenic ligands. Proc. Natl. Acad. Sci. USA.

[218] Ma C., Fan R., Ahmad H., Shi Q., Comin-Anduix B., Chodon T., Koya R.C., Liu C.-C., Kwong G.A., Radu C.G. (2011). A clinical microchip for evaluation of single immune cells reveals high functional heterogeneity in phenotypically similar T cells. Nat. Med..

[219] McWhorter F.Y., Smith T.D., Luu T.U., Rahim M.K., Haun J.B., Liu W.F. (2016). Macrophage secretion heterogeneity in engineered microenvironments revealed using a microwell platform. Integr. Biol. Quant. Biosci. Nano Macro.

[220] An X., Sendra V.G., Liadi I., Ramesh B., Romain G., Haymaker C., Martinez-Paniagua M., Lu Y., Radvanyi L.G., Roysam B. (2017). Single-cell profiling of dynamic cytokine secretion and the phenotype of immune cells. PLoS ONE.

[221] Chalaris A., Garbers C., Rabe B., Rose-John S., Scheller J. (2011). The soluble Interleukin 6 receptor: Generation and role in inflammation and cancer. Eur. J. Cell Biol..

[222] Meager A. (2006). Measurement of cytokines by bioassays: Theory and application. Methods.

[223] Kovarik P., Ebner F., Sedlyarov V. (2017). Posttranscriptional regulation of cytokine expression. Cytokine.

